# Plant microRNAs in larval food regulate honeybee caste development

**DOI:** 10.1371/journal.pgen.1006946

**Published:** 2017-08-31

**Authors:** Kegan Zhu, Minghui Liu, Zheng Fu, Zhen Zhou, Yan Kong, Hongwei Liang, Zheguang Lin, Jun Luo, Huoqing Zheng, Ping Wan, Junfeng Zhang, Ke Zen, Jiong Chen, Fuliang Hu, Chen-Yu Zhang, Jie Ren, Xi Chen

**Affiliations:** 1 State Key Laboratory of Pharmaceutical Biotechnology, NJU Advanced Institute for Life Sciences, Jiangsu Engineering Research Center for MicroRNA Biology and Biotechnology, Nanjing University, Nanjing, China; 2 College of Animal Science, Zhejiang University, Hangzhou, China; 3 Model Animal Research Center and MOE Key Laboratory of Model Animals for Disease Study, Nanjing University, Nanjing, China; 4 Cold Spring Harbor Laboratory, Cold Spring Harbor, NY, United States of America; The University of North Carolina at Chapel Hill, UNITED STATES

## Abstract

The major environmental determinants of honeybee caste development come from larval nutrients: royal jelly stimulates the differentiation of larvae into queens, whereas beebread leads to worker bee fate. However, these determinants are not fully characterized. Here we report that plant RNAs, particularly miRNAs, which are more enriched in beebread than in royal jelly, delay development and decrease body and ovary size in honeybees, thereby preventing larval differentiation into queens and inducing development into worker bees. Mechanistic studies reveal that *amTOR*, a stimulatory gene in caste differentiation, is the direct target of miR162a. Interestingly, the same effect also exists in non-social *Drosophila*. When such plant RNAs and miRNAs are fed to *Drosophila* larvae, they cause extended developmental times and reductions in body weight and length, ovary size and fecundity. This study identifies an uncharacterized function of plant miRNAs that fine-tunes honeybee caste development, offering hints for understanding cross-kingdom interaction and co-evolution.

## Author summary

How caste has formed in honeybees is an enduring puzzle. The prevailing view is that royal jelly stimulates the differentiation of larvae into queen. Here, we uncover a new mechanism that plant miRNAs in worker bee’s food postpone larval development, thereby inducing sterile worker bees. Thus, the theories about honeybee caste formation need to be re-examined from a new angle besides the traditional focus on royal jelly and its components. Furthermore, since miRNAs are transmitted between species of different kingdoms and can contribute to the phenotype regulation, this new model of horizontal miRNA transfer may open up a new avenue to further study the molecular mechanisms underlying cross-kingdom interaction and co-evolution.

## Introduction

Caste development in social insects represents a major transition from one level of organization to another in evolution and is believed to be central to the ecological success of social insects [1]. How castes evolved is an enduring puzzle that has long fascinated scientists but currently has no satisfactory answers. Honeybees (*Apis mellifera*) represent a principal example of caste development. Female honeybees develop into two castes, queens and workers, which differ in morphology, physiology and social function [1, 2]. The queens are reproductive, have a larger body size, develop faster and live longer, whereas workers are characterized by the opposite traits and are mostly sterile helpers that nourish larvae and collect food [3]. This dimorphism is not a consequence of genetic differences but is mainly determined by larval feeding: female larvae receiving a rich diet of royal jelly develop into queens, whereas a less sophisticated diet named “beebread” leads to the worker bee fate [4, 5]. However, it is still not fully understood how different diets modify the developmental trajectory of honeybees to such a thorough extent. While several components of the larval diet, such as specific royal jelly proteins, sugars, p-coumaric acid and fatty acids, have been independently shown to influence caste development in honeybees [6–10], they still cannot account for the full impact of larval food on honeybee development. In this study, we investigated a largely overlooked component of larval food, microRNA (miRNA), and examined its effect on caste development.

miRNAs are a class of 19–24-nucleotide-long non-coding RNAs that act as post-transcriptional regulators of gene expression in eukaryotes [11]. Recently, we reported an unexpected finding that plant miRNAs that are ingested from plant food sources can pass through the gastrointestinal tract, enter into the blood, accumulate in tissues and regulate endogenous gene expression in animals [12]. Other studies have also documented the importance of small RNAs that are transmitted from one species to another and facilitate cross-talk and interspecies communication [13–16]. Moreover, multiple studies have proven that dietary exogenous miRNAs are detectable in consumed animal blood and tissues [17–20]. These studies furnish an additional layer of gene regulation: cross-kingdom regulation mediated by exogenous miRNAs. It is very tempting to speculate that small RNAs in larval food may be an active component that influences honeybee development.

Because beebread is a mixture of pollen and honey, while royal jelly is a glandular secretion of nurse bees [4], the main food sources of worker- and queen-destined larvae are, in theory, plant- and animal-derived, respectively. Thus, we hypothesize that different miRNA contents from larval food of different origins may have distinct impacts on honeybee development. In agreement with this hypothesis, it has been well established in the literature that insects, including honeybees and fruit flies, can ingest small RNAs and that ingested small RNAs can regulate the expression of insect genes, thus reshaping the insects’ phenotypes [21–24]. In this study, we provide evidence for a previously uncharacterized regulatory mechanism of worker bee development, which can be partially attributed to the plant miRNAs enriched in beebread and pollen fed to young larvae.

## Results

### Plant miRNAs are more enriched in beebread and pollen than in royal jelly

**Fig 1:**
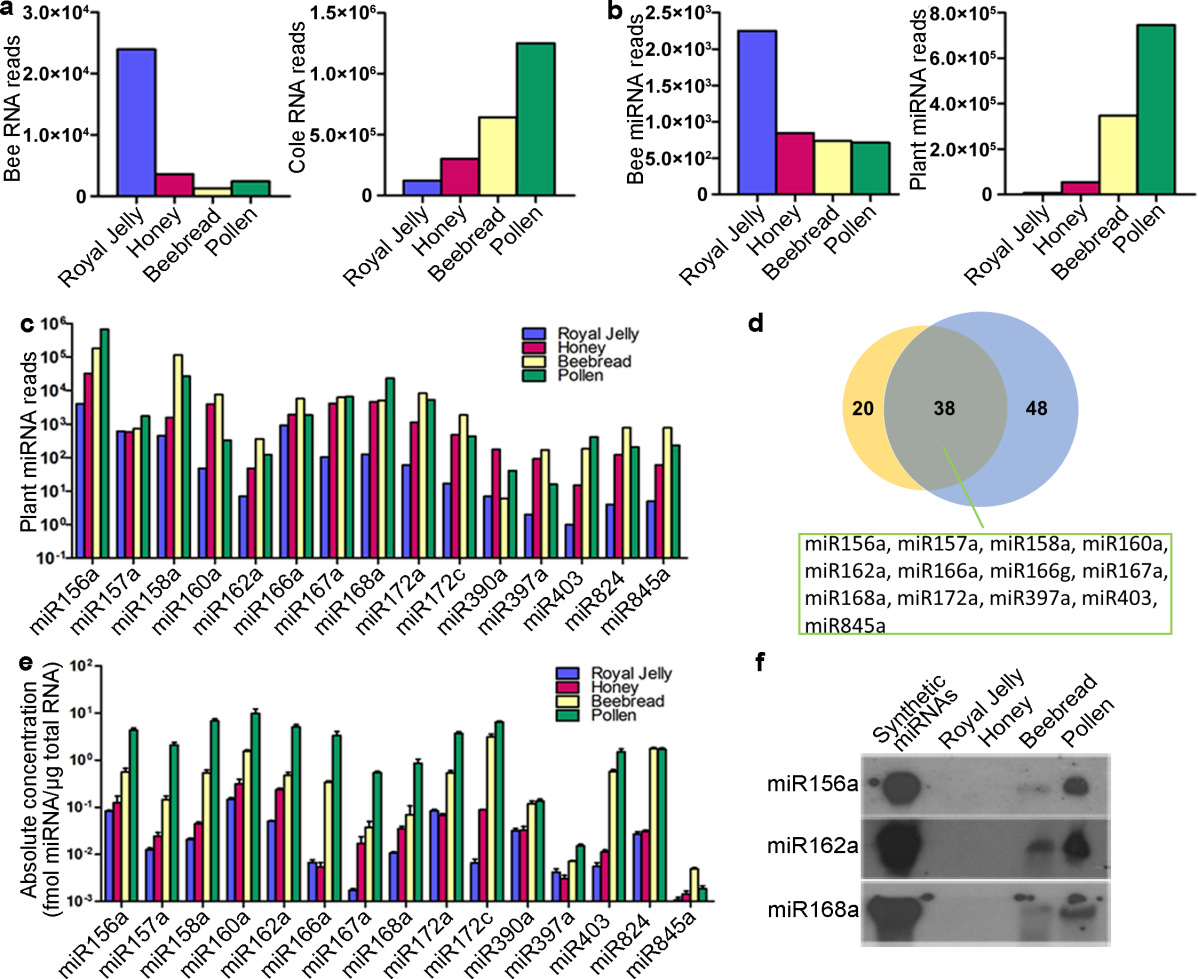
Comparison of the levels of plant and animal miRNAs in royal jelly, honey, beebread and pollen. (**a**) The levels (total sequencing reads) of bee and cole RNAs as detected via Illumina deep-sequencing in royal jelly, honey, beebread and pollen collected during cole flowering stage. (**b**) The levels (total sequencing reads) of bee and plant miRNAs as detected via Illumina deep-sequencing in royal jelly, honey, beebread and pollen collected during cole flowering stage. (**c**) The levels (sequencing reads) of 16 representative plant miRNAs in royal jelly, honey, beebread and pollen collected during cole flowering stage. (**d**) Number and overlap of plant miRNAs in beebread collected during cole (yellow) and camelia (blue) flowering stage. For the 16 representative plant miRNAs, 13 are present in both cole and camellia pollen. (**e**) The absolute levels of 16 representative plant miRNAs as detected via qRT-PCR in royal jelly, honey, beebread and pollen collected during cole flowering stage. miRNA levels were normalized to the total amounts of RNA. Data are represented as the mean ± SEM. Note that the qRT-PCR primer for miR166g was not commercially available, therefore only 15 representative plant miRNAs were assessed. (**f**) Northern blotting analysis of the levels of miR156a, miR162a and miR168a in royal jelly, honey, beebread and pollen collected during cole flowering stage. Synthetic miRNAs served as positive controls. Equal amounts of total RNA (15 μg) were loaded for northern blotting.

First, we analysed the small RNA components in royal jelly, honey, beebread and pollen using Illumina deep-sequencing technology. To investigate pollen as a larval food source under natural conditions, we used bee pollen collected and packed by worker bees. The royal jelly, honey, beebread and pollen were collected during the cole (*Brassica campestris*) flowering stage. Consistent with previous reports [25, 26], the lengths of small RNAs in pollen were concentrated in a range from 19 to 24 nucleotides (S1 Fig). However, the lengths of small RNAs in royal jelly, honey and beebread were distributed over a wider range, from 13 to 28 nucleotides, probably due to degradation products from longer RNAs during their processing within the beehive. Next, total small RNAs were mapped to the reference transcriptome assemblies of honeybee and cole and were further assigned to different classes of small RNAs. In agreement with the hypothesis that royal jelly RNA is mainly animal-derived and beebread RNA is plant-derived, honeybee small RNAs were present at a far higher level in royal jelly than in beebread and pollen, while the abundance of cole small RNAs gradually increased from royal jelly to honey to beebread and pollen (Fig 1A).

A large proportion of the small RNAs were annotated as miRNAs and as the degradation products of tRNAs, rRNAs and mRNAs. By aligning small RNA reads to known miRNAs in the miRBase database 21.0, a total of 46, 39, 14 and 15 annotated bee miRNA types were detected in royal jelly, honey, beebread and pollen, respectively (S1 Table). Most of the bee miRNAs had less than 10 sequence reads in the samples, but they had much higher reads in royal jelly than in honey, beebread and pollen (Fig 1B). On the other hand, there were 41, 71, 58 and 53 annotated plant miRNA types in royal jelly, honey, beebread and pollen, respectively (S1 Table). These plant miRNAs were present at far higher concentrations than animal miRNAs, and their concentration in beebread and pollen was invariably much higher than that in royal jelly and honey (Fig 1B). The differential enrichment of plant miRNAs in beebread and animal miRNAs in royal jelly is clearly shown in S2A Fig. In contrast, the miRNA compositions of beebread and pollen showed high similarities to each other, with a Pearson’s correlation coefficient (R) close to 1 (S2A Fig). The 16 representative plant miRNAs (miR156a, miR157a, miR158a, miR160a, miR162a, miR166a, miR166g, miR167a, miR168a, miR172a, miR172c, miR390a, miR397a, miR403, miR824 and miR845a) with the highest concentrations in beebread and pollen of cole are listed in Fig 1C.

Given the diversity of pollen that is collected by honeybees, plant miRNAs might not be uniformly present in pollen from different sources. Therefore, it is essential to analyze the small RNA components in beebread and pollen collected from different geographical and botanical sources. We performed deep sequencing on royal jelly, honey, beebread and pollen collected during the camellia (*Camellia japonica*) flowering stage. The results revealed again that the plant miRNAs were more abundant in pollen and beebread than in royal jelly and honey (S2 Table). Likewise, the miRNA profiles were quite similar between beebread and pollen and widely different between beebread and royal jelly (S2B Fig). Interestingly, the plant miRNA profiles of cole and camellia beebread showed similarity to each other, especially for many plant miRNAs that are evolutionarily conserved across the major lineages of plants. For example, 13 of the 16 plant miRNA species enriched in cole beebread were also present in camellia beebread (Fig 1D). Thus, the global components of plant miRNAs in beebread and pollen may not be very diverse between different sources.

However, because deep sequencing is inferior to the more commonly used qRT-PCR for miRNA quantification [27], we performed qRT-PCR assays with a standard curve set using synthetic oligonucleotides of known concentrations to determine the actual concentrations of plant miRNAs in royal jelly, honey, beebread and pollen. All 16 representative plant miRNAs except miR166g (whose qRT-PCR primer was not commercially available) could be readily detected using qRT-PCR in beebread and pollen of cole but were nearly undetectable in royal jelly and honey (generally < 0.1 fmol per μg total RNA) (Fig 1E). It should be noted that we used two normalization strategies for cross-sample comparisons of miRNAs in royal jelly and beebread, and both strategies showed that each plant miRNA was much more abundant in beebread than in royal jelly (S2C Fig). Moreover, northern blotting, which can determine the sizes and concentrations of RNAs, produced the same differences described above for plant miRNA concentrations and showed that miR156a, miR162a and miR168a were detectable in beebread and pollen but not in royal jelly and honey (Fig 1F).

### Effects of plant RNA on honeybee development

To investigate the effects of plant RNAs, and particularly miRNAs, on honeybee phenotypes, we removed the larvae from the colony setting and reared them on a laboratory diet with or without the addition of plant RNAs or miRNAs. To avoid overfeeding and generating supra-physiological effects, our pilot study first determined the amounts of the 16 representative plant miRNAs that were contained in natural beebread (Fig 1E and S2C Fig). Since the plant miRNA composition enriched in natural beebread is very similar to that in pollen (S2A Fig and S2B Fig), we added total RNA purified from cole pollen to the laboratory diet at the same level as determined based on miRNA levels to reconstitute a close mimic of natural beebread in terms of its miRNA components (“beebread mimic” in S3 Fig). When developing larvae were fed with this beebread mimic, 2-fold of beebread mimic dramatically suppressed the growth of the developing larvae and even caused some larvae to die, whereas 0.5- or 1-fold of beebread mimic reduced larvae growth but had little effect on their survival (S4 Fig).

**Fig 2:**
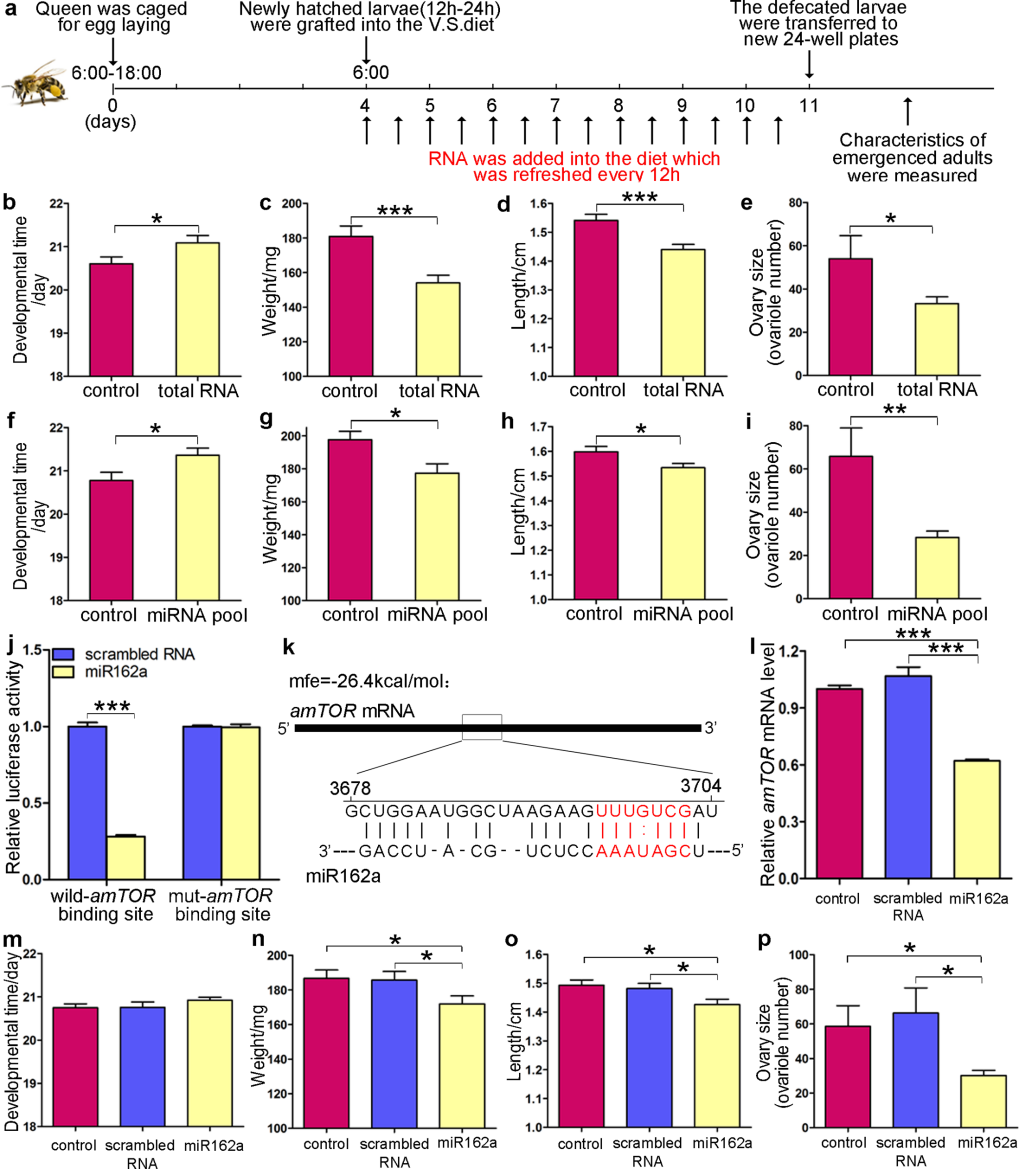
Effects of plant RNAs, plant miRNA pool and miR162a on honeybee phenotypes. (**a**) Flow chart of the experimental design. (**b-e**) Developmental time (b), body weight (c), length (d) and ovary size (e) of honeybees that were reared with the control diet (equal volume of DEPC water added) or a diet containing total pollen RNA (n = 25–30). Data are represented as the mean ± SEM. *p < 0.05; ***p < 0.001, Student’s *t*-test. (**f-i**) Developmental time (f), body weight (g), length (h) and ovary size (i) of honeybees that were reared with the control diet or a diet supplemented with the synthetic miRNA pool (n = 25–30). Data are represented as the mean ± SEM. *p < 0.05; **p < 0.01, Student’s *t*-test. (**j**) Firefly luciferase reporters containing the potential binding site or mutant binding site for miR162a in the *amTOR* gene were co-transfected with scrambled RNAs or miR162a into 293T cells. At 24 h post-transfection, cells were assayed using luciferase assay kits. Data are represented as the mean ± SEM. ***p < 0.001, Student’s *t*-test. (**k**) Schematic illustration of the hypothetical duplex formed by the interaction between *amTOR* (top) and miR162a (bottom) in honeybees. The predicted free energy of the hybrid is indicated. (**l**) qRT-PCR analysis of the levels of *amTOR* mRNA in 4^th^ instar honeybee larvae reared with control diets or diets supplemented with synthetic miR162a. Error bars represent SEM. ***p < 0.001, one-way ANOVA. (**m-p**) Developmental time (m), body weight (n), length (o) and ovary size (p) of honeybees reared with the control diet or a diet supplemented with synthetic scrambled RNA or miR162a (n = 25–30). Data are represented as the mean ± SEM. *p < 0.05, one-way ANOVA.

Next, the effects of plant RNA supplements were characterized based on the developmental time, weight, length and ovary size of adult bees immediately upon emerging from the pupal stage (Fig 2A). Feeding larvae with beebread mimic increased the whole-body accumulation of the 16 representative plant miRNAs (S5A Fig). We did not distinguish the particular tissues where the ingested plant miRNAs were located but instead investigated the effects of plant miRNAs on the whole body as the uptake of exogenous small RNAs from the insect gut has been frequently observed [21, 22, 28]. As a result of the plant RNA supplements, larvae grew relatively slowly during their development and emerged as adults with more of a worker morphology (S6 Fig) characterized by a prolonged developmental time (on average 0.49 days longer, p = 0.0444), reduced weight (on average 14.81% lighter, p = 0.0008) and size (on average 6.55% shorter, p = 0.0005) at adult emergence and a decreased ovary size (on average 21 fewer ovarioles, p = 0.0358) (Fig 2B–2E).

### Effects of a plant miRNA pool on honeybee phenotypes

To validate the contribution of plant miRNAs to the observed honeybee phenotypes, we synthesized the 16 plant miRNAs enriched in beebread and pollen, and then the synthetic miRNA pool was added to the larval diet at levels equivalent to those in natural beebread. Compared to the control group, honeybees that were fed a diet containing the miRNA pool showed an increased accumulation of corresponding plant miRNAs within their body (S5B Fig) and developed worker bee-like characteristics, i.e., reduced sizes at adult emergence (10.27% lighter and 4.01% shorter, p = 0.0194 and p = 0.0264, respectively), extended pre-adult developmental time (0.58 days longer, p = 0.0254) and decreased ovary sizes (38 fewer ovarioles, p = 0.0094) (Fig 2F–2I).

### miR162a acts on *amTOR* to induce worker bee phenotypes

Next, we performed bioinformatics analysis to dissect the potential functions of the plant miRNAs in honeybee’s food. Two bioinformatic algorithms (RNAhybrid and miRanda) were used in combination to scan honeybee mRNA sequences for potential binding sites for the 16 representative plant miRNAs. A total of 96 honeybee genes were predicted by both RNAhybrid and miRanda algorithms as the target genes of the 16 plant miRNAs. Most of the 96 genes were predicted to be targeted by only one plant miRNA, whereas a few genes were common targets of 2–3 plant miRNAs. We then used Gene Ontology (GO) analysis to look for biological processes that might be associated with the 96 target genes of the 16 plant miRNAs based on the strategy of a previous study [29]. Significant enrichment of GO functional categories related to “development” was observed (S7 Fig and S3 Table), suggesting again that the plant miRNAs specifically enriched in beebread and pollen may be involved in regulation of the development process of honeybees. Among the 96 target genes, some genes known to influence the developmental fate of honeybees were specially selected and listed in S4 Table.

Subsequently, plant miRNAs targeting *Apis mellifera* TOR (amTOR) were analysed, as previous studies have demonstrated that *amTOR* plays a stimulatory role in caste development: the queen fate is associated with elevated *amTOR* activity, and the inhibition of *amTOR* causes developmental changes towards worker characteristics in queen-destined larvae [22, 30, 31]. To screen for plant miRNAs that could directly regulate *amTOR* expression, luciferase reporter assays were conducted. Each plant miRNA binding site in the *amTOR* gene was fused separately into a position downstream of the firefly luciferase gene in a reporter plasmid. The resulting plasmids were co-transfected into a cell line in combination with above-mentioned plant miRNAs. Among 9 plant miRNAs that could potentially target amTOR, miR162a resulted in a 72% decrease in luciferase activity (Fig 2J), whereas miR156a showed a 14% reduction and other 7 plant miRNAs did not affect luciferase activity (S8 Fig), suggesting that miR162a specifically recognizes *amTOR* and mediates the post-transcriptional inhibition of this gene. In addition, the *amTOR*/miR162a hybrid is illustrated in Fig 2K, and its free energy was -26.4 kcal/mol, which was well within the ranges of genuine miRNA-target pairs (-17 kcal/mol is a cutoff value of free energy) [32]. However, when point mutations were introduced into the predicted “seed site” in the *amTOR* gene, the fused luciferase reporters were no longer affected by miR162a (Fig 2J).

Subsequently, to determine the potential effects of miR162a alone on *amTOR* expression and the corresponding phenotypes, honeybee larvae were reared with a diet to which either synthetic miR162a or scrambled RNA was added. Notable increases in the amount of ingested miR162a (S5C Fig) and decreases in the level of *amTOR* mRNA (Fig 2L) were detected in honeybees that were reared with a diet containing miR162a. Similarly, *amTOR* mRNA was downregulated in honeybees reared on a diet containing either total pollen RNA or the synthetic miRNA pool (S9A and S9B Fig). In contrast to the scrambled RNA, which had no effect on any of the tested morphological characteristics, miR162a supplied in the larval food significantly reduced the body weights (7.87% lighter, p = 0.0292) and lengths (4.49% shorter, p = 0.0103) and ovary sizes (29 fewer ovarioles, p = 0.0301) of newly emerged adults but did not significantly increase the developmental time (0.17 days longer, p = 0.1755) of the adult bees (Fig 2M–2P).

### Effects of plant RNA, the plant miRNA pool and miR162a on *Drosophila* phenotypes

**Fig 3:**
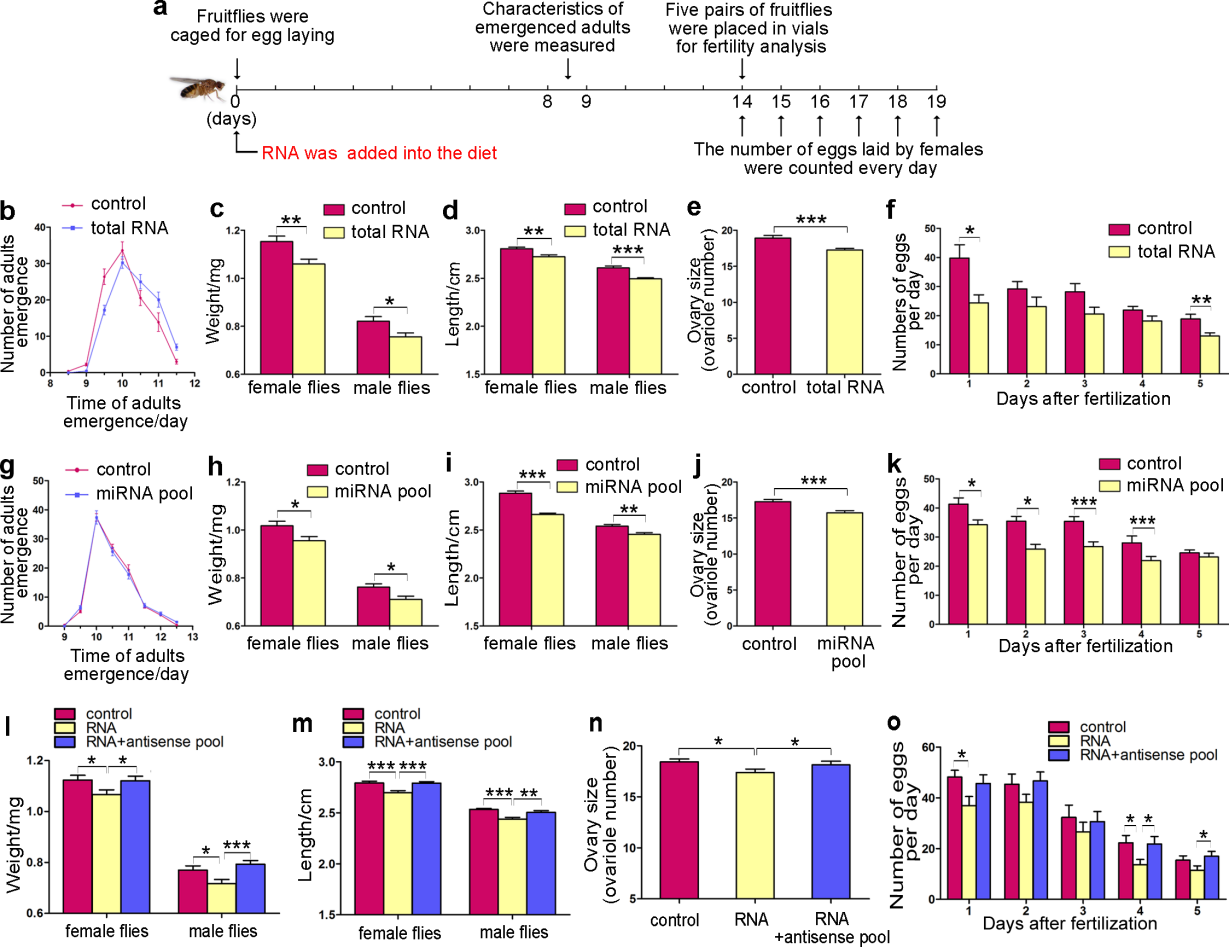
Effects of plant RNA and plant miRNA pool on *Drosophila* phenotypes. (**a**) Flow chart of the experimental design. (**b-f**) Developmental time (b), body weight (c), body length (d), ovary size (e) and fecundity (f) of *Drosophila* reared with control medium (equal volume of DEPC water added) or medium containing total pollen RNA (n = 25–35). Data are represented as the mean ± SEM. *p < 0.05; **p < 0.01; ***p < 0.001, Student’s *t*-test. (**g-k**) Developmental time (g), body weight (h), body length (i), ovary size (j) and fecundity (k) of *Drosophila* reared with control medium or medium supplemented with the synthetic miRNA pool (n = 25–35). Data are represented as the mean ± SEM. *p < 0.05; **p < 0.01; ***p < 0.001, Student’s *t*-test. (**l-o**) Body weight (l), body length (m), ovary size (n) and fecundity (o) of *Drosophila* reared with control medium or medium supplemented with pollen RNA or with pollen RNA plus a plant antisense miRNA pool (n = 25–35). Data are represented as the mean ± SEM. *p < 0.05; **p < 0.01; ***p < 0.001, one-way ANOVA.

To further investigate the evolutionary dynamics of the molecular mechanisms underlying social development between solitary and eusocial species, we tested plant RNA and miRNAs on a non-social model insect, *Drosophila melanogaster*. Although there is no caste differentiation in *Drosophila*, there is evidence that molecular pathways involved in establishing caste dimorphism are also conserved in the individual development of *Drosophila* [6]. Thus, we investigated the mechanism underlying honeybee caste differentiation in *Drosophila* (Fig 3A).

First, we ruled out the possibility that the residual chemicals from RNA isolation might block larval development as the mock group of *Drosophila* larvae fed the same chemical residues developed normally (S10 Fig). In accordance with the observation that the beebread mimic postpones queen differentiation in honeybees, *Drosophila* larvae reared with medium containing total pollen RNA had longer developmental times (p<0.0001), were smaller (7.83% lighter and 2.99% shorter in females, p = 0.0038 and p = 0.0014, respectively; 7.32% lighter and 4.33% shorter in males, p = 0.0144 and p<0.0001, respectively), had fewer ovarioles (1.6 fewer ovarioles, p = 0.0008) and showed reduced fecundity (a total of 28.04% eggs fewer) compared to those reared with the control medium (Fig 3B–3F). Similarly, plant miRNAs also accumulated in *Drosophila* larvae (S5D Fig). Subsequently, to narrow down the active components in plant RNA, small RNAs were enriched from total pollen RNA, and the effects of small RNAs on *Drosophila* phenotypes were examined in the same manner as described above. Small plant RNAs also delayed *Drosophila* development (p<0.0001) and reduced the final adult size (6.84% lighter and 3.45% shorter in females, p = 0.0002 and p = 0.0005, respectively; 7.50% lighter and 3.98% shorter in males, p = 0.0030 and p = 0.0010, respectively), ovary size (0.9 fewer ovarioles, p = 0.0288) and fecundity (25.13% eggs fewer) of *Drosophila* as effectively as total RNA (S11 Fig).

Similarly, when the miRNA pool was fed to developing *Drosophila* larvae, we observed an increase in plant miRNA levels (S5E Fig) and corresponding decreases in final adult size (5.88% lighter and 7.73% shorter in females, p = 0.0208 and p<0.0001, respectively; 6.82% lighter and 3.39% shorter in males, p = 0.0111 and p = 0.0013, respectively), ovary size (1.6 fewer ovarioles, p = 0.0004) and fecundity (19.96% eggs fewer) (Fig 3H–3K). However, developmental times did not change in *Drosophila* reared with medium containing the miRNA pool (p = 0.768) (Fig 3G). To determine the specificity of the inhibitory effects of plant miRNAs on *Drosophila* development and to exclude the possibility that the phenotypic changes were caused by components other than plant miRNAs, an miRNA antisense pool against the above-mentioned 16 miRNAs was synthesized and added to the *Drosophila* larval medium together with small pollen RNAs to abolish the function of these plant miRNAs. The inhibitory effects of plant RNAs on the adult size, ovary size and fecundity of *Drosophila* were completely reversed by the addition of the antisense pool to the larval diet (Fig 3L–3O).

**Fig 4:**
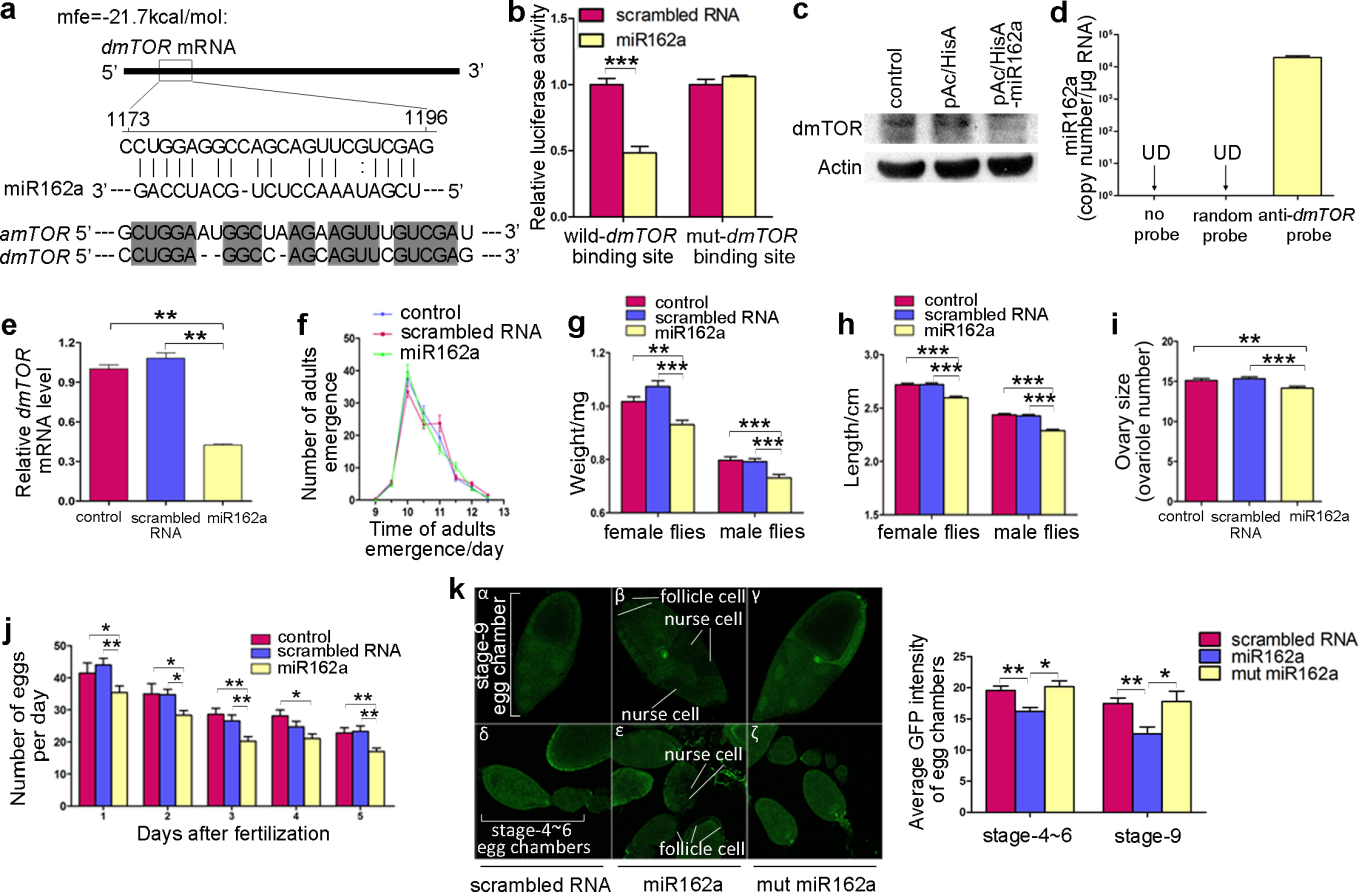
Effects of miR162a on *dmTOR* gene expression and *Drosophila* phenotypes. (**a**) Schematic illustration of the hypothetical duplex formed by the interaction between *dmTOR* (top) and miR162a (bottom) in *Drosophila*. The predicted free energy of the hybrid is indicated. The sequences in the binding site are highly conserved across honeybees and *Drosophila*. (**b**) Firefly luciferase reporters containing the potential binding site or a mutant binding site for miR162a in the *dmTOR* gene were co-transfected with scrambled RNA or miR162a into 293T cells. At 24 h post-transfection, cells were assayed using luciferase assay kits. Data are represented as the mean ± SEM. ***p < 0.001, Student’s *t*-test. (**c**) Western blotting analysis of the levels of dmTOR protein in control S2 cells or in S2 cells transfected with an empty pAc5.1 vector or with a pAc5.1 vector expressing miR162a. For the expression of miR162a, a fragment containing the miR162a sequence was cloned into the pAc5.1 vector. The image is a representative of three biological replicates. (**d**) The absolute levels of miR162a in the pull-down products precipitated by the anti-*dmTOR* probe, random probe or no probe were assessed via qRT-PCR. UD, undetectable. (**e**) qRT-PCR analysis of the levels of *dmTOR* mRNA in 3-day-old *Drosophila* larvae reared with control medium or medium supplemented with synthetic miR162a. Error bars represent SEM. **p < 0.01, one-way ANOVA. (**f-j**) Developmental time (f), body weight (g), body length (h), ovary size (i) and fecundity (j) of *Drosophila* reared with control medium or medium supplemented with synthetic scrambled RNA or miR162a (n = 25–35). Data are represented as the mean ± SEM. *p < 0.05; **p < 0.05; ***p < 0.001, one-way ANOVA. (**k**) Decrease in GFP levels in the egg chambers of pUbi-GFP-*dmTOR* transgenic *Drosophila* when miR162a was added to the larval diet. The miR162a binding sequence in the *dmTOR* gene was fused downstream of the GFP reporter gene in a pUbi-GFP expression vector, and a pUbi-GFP-*dmTOR* transgenic *Drosophila* line was created via embryo injection. Differences in GFP expression in nurse cells and follicle cells of stage-9 egg chambers (α-γ) or stage-4~6 egg chambers (δ-ζ) were observed when miR162a or mutant miR162a was added to the larval diet of the transgenic line, respectively. Left panel: representative images; Right panel: quantitative analysis (n = 12).

Next, a similar miR162a binding site in the *Drosophila melanogaster* TOR (*dmTOR*) gene was identified (Fig 4A). When this binding site was fused into the luciferase reporter plasmid, miR162a also reduced luciferase activity (Fig 4B). However, when a point mutation was introduced into the miR162a binding site in the *dmTOR* gene, the mutated luciferase reporter was unaffected by miR162a (Fig 4B). The correlation between miR162a and *dmTOR* was further examined by evaluating dmTOR protein expression in *Drosophila* Schneider 2 cells (S2 cells) after the induction of miR162a. The expression of the dmTOR protein was significantly inhibited by miR162a in S2 cells (Fig 4C). We further performed a biotin-avidin pull-down assay to assess the direct binding of miR162a to *dmTOR* mRNA. miR162a was only enriched in the pull-down product precipitated by the anti-*dmTOR* probe and was undetectable in the products that were precipitated by a random probe or no probe (Fig 4D), suggesting that miR162a directly binds to *dmTOR* mRNA in S2 cells.

Moreover, *Drosophila* larvae reared with synthetic miR162a supplied in the medium showed increased whole-body accumulation of miR162a (S5F Fig) and reduced whole-body expression of *dmTOR* mRNA (Fig 4E). A similar reduction in *dmTOR* mRNA levels was observed in *Drosophila* reared with medium containing total pollen RNA or the synthetic miRNA pool (S9C and S9D Fig). Consequently, *Drosophila* reared with miR162a in the medium exhibited a decrease in body weight (8.82% and 8.75% lighter in females and males, p = 0.0013 and p = 0.0003, respectively), length (4.45% and 6.04% shorter in females and males, p<0.0001 and p<0.0001, respectively), ovary size (0.9 fewer ovarioles, p = 0.0050) and fecundity (21.79% eggs fewer) but had equal developmental times compared to the control larvae (p = 0.323) (Fig 4F–4J). In contrast, *Drosophila* larvae reared with the scrambled RNA in the medium showed no such phenotypes.

Finally, the correlation between miR162a and *dmTOR* was analysed using transgenic *Drosophila* expressing a GFP reporter transgene with an miR162a binding fragment of *dmTOR* inserted downstream. In association with the observed phenotype of reduced fecundity in *Drosophila* reared with miR162a in the medium (Fig 4J), decreased GFP levels in both nurse cells and follicle cells in the egg chambers were observed when miR162a was added into the larval diets of the transgenic line, while the addition of scrambled or seed-mutant miR162a mimics had no effect on GFP levels (Fig 4K). These results suggest that miR162a in larval food was sufficiently delivered to *Drosophila* ovaries and that it suppresses endogenous *dmTOR* expression.

## Discussion

Caste differentiation of honeybees is a complex developmental process influenced by genetic, epigenetic and environmental variations. The prevailing view is that the nutrients in royal jelly (primarily proteins, sugars and fatty acids) drive queen development [6, 10, 33, 34]. However, the active components that determine the developmental fate of honeybees remain elusive and even controversial [35]. Recent studies provide new insights into the relationship between epigenetic regulation and caste differentiation in insects [36–38]. In this study, we identified that plant miRNAs are significantly enriched in beebread and pollen and not in royal jelly. This striking difference prompted us to hypothesise that miRNAs, acting as important epigenetic regulators, may be transferred from the food of worker-destined larvae to their bodies and negatively regulate larval development; in contrast, miRNAs in the royal jelly are not sufficient to reach a functional level and to have biological relevance, therefore queen-destined larvae consuming royal jelly evade miRNA regulation. To test this hypothesis, we elucidated the effects of the plant RNAs and miRNAs that were enriched in beebread and pollen on honeybee phenotypes, and uncovered a previously unrecognized role for RNA as an environmental determinant of honeybee caste development. Furthermore, we investigated phenotypic changes in *Drosophila* caused by food supplemented with plant RNAs and miRNAs and observed larvae developing into adults with phenotypes similar to those of worker bees. We verified that these effects on the development of honeybees and *Drosophila* were caused by plant RNAs and specific miRNAs and excluded the possibility of a general effect of RNAs, because a synthetic scrambled RNAs added to the larval diet did not cause any phenotypic changes in honeybees or *Drosophila*. We also ruled out the possibility that the potentially toxic effects of chemical residues from the RNA isolation process caused the observed phenotypic changes in *Drosophila*, because a mock diet (H_2_O instead of pollen was processed for RNA isolation and added to the diet) with similar chemical residues had no effects on larval development. As a next step, we investigated whether honeybee development was regulated by variations in specific genes that are targeted by specific plant miRNAs. Mechanistic studies revealed that the blocking of the queen fate was, at least in part, due to *amTOR* knockdown by miR162a. Overall, our study revealed that the development of worker bee caste may be, at least in part, attributed to a previously uncharacterized effect executed by the transfer of enriched plant miRNAs in beebread and pollen to the young larvae.

The mobility of small RNA molecules (e.g., siRNA and miRNA) from one species to another is a newly discovered mechanism for the spread of gene-silencing signals and for facilitating cross-talk between different organisms, even between species of different kingdoms [39]. The cross-species transfer of small RNAs has been frequently reported to occur between interacting organisms: from bacteria to nematodes [40], from fungal pathogens to plants [14], from plants to pathogenic and symbiotic microbes [41–44], from plants to nematodes [45], and from plants to insects [46]. For example, transgenic plants engineered to produce siRNAs against essential pest genes are more resistant to pest attack [46]. In this study, we sought to broaden the understanding of the existence of small RNA transfer between representative species in the natural world: honeybees and plants. Our evidence indicates that ingested plant miRNAs affect gene expression and can reshape honeybee phenotypes, and it may provide additional support for the concept of horizontal small RNA transfer. We focused on the phenomenon of plant miRNA uptake and function but did not uncover a clear molecular mechanism accounting for the entrance and transfer of miRNAs within honeybees. We propose that systemic RNAi, which allows small RNAs to be transported across cellular boundaries and to spread throughout the whole body of insects [21, 22, 28], might be a possible transport mechanism. However, this mechanism, which is mediated through SID-1 transmembrane protein activity [47, 48], has only been intensively characterized in *C*. *elegans*. Whether SID-1 homologues are present in honeybees and play equivalent roles in small RNA transport requires further investigation. Another open question is how honeybees make use of the available dosage of plant miRNAs to control their development. In our experiments, the same amount of plant miRNAs as is found in natural beebread was used, and this dose produced similar effects to those seem in nature. It is largely unknown if honeybees possess an amplification pathway as is found in *C*. *elegans* [49] to allow a small amount of RNA taken up from the environment to generate abundant secondary RNAs and to trigger strong responses within the body. In addition, plant miRNAs tend to induce mRNA cleavage through perfect or near-perfect complementarity with their target sequences, while animal miRNAs generally cause translational repression through partial complementarity [11, 50–52]. The observation that miR162a decreased *amTOR* mRNA levels *in vivo* indicates that it behaves, at least in some ways, similarly to a plant miRNA. However, miR162a shows non-perfect complementarity with its target sequence, even with a G:U wobble in the seed region, indicating a regulatory action of animal miRNA. It is also unclear how plant miRNAs are incorporated into the honeybee’s Argonaute complexes. Because the ingested plant miRNAs should be mature single-stranded RNAs, it is not clear how these single-stranded small RNAs are loaded into Argonaute proteins to produce a functional miRNA form. Nevertheless, because miRNAs and other small RNAs have been frequently detected to be transported between species and hijack the RNAi machinery of host cells to exert biological functions [14, 40–44], it would be interesting to analyse the mode of action of plant miRNAs in honeybee cells. However, these questions are beyond the scope of this study.

Protocols have been developed for rearing honeybee since 1927 [53–55]. The diet of a mixture of fresh royal jelly, fructose, glucose, yeast extract and H_2_O has been proven to be the optimal food for honeybee larvae [56–58]. In this study, it should be noted that one direct test could be to feed honeybees with beebread in which plant miRNAs have been eliminated. In fact, we have attempted to rear honeybees with pollen or beebread supplemented with antisense miRNAs without royal jelly. Unfortunately, all of the larvae died during cultivation. This result is consistent with previous observations that royal jelly is indispensable for the rearing of honeybee larvae *in vitro* [54, 55, 59]. Alternatively, we added plant RNAs or miRNAs to the larval diet of honeybees, which can defer the queen bee fate even in the presence of royal jelly and therefore supports our arguments. In fact, queen development is not the default trajectory in honeybees and royal jelly is needed to act on the endocrine system to direct larvae differentiation into a queen fate. The pathways controlling body size, developmental duration and fertility are anyway downregulated in worker-destined larvae [22, 30, 31]. According to our study, we suggest that the negative effects of beebread and pollen on larval development may be a part of the causation. Additionally, lab-reared honeybees largely develop with intermediate characteristics between a worker and queen, i.e., with more ovarioles than natural worker bees [60]. This phenomenon implies that an essential ingredient may be missing from the larval diet used for *in vitro* cultivation that impairs the differentiation of worker bees. We suggest that the plant RNA enriched in natural beebread is a very likely candidate, although we cannot rule out other possibilities.

Caste development is a complex process that involves multiple regulatory factors. Although this study largely focused on how plant miRNAs negatively affect the development of honeybees, we do not claim that plant miRNAs are the sole factor regulating honeybee development, and thus, removing plant miRNAs alone is not sufficient to disrupt the development of all phenotypes related to caste differentiation. Likewise, we do not expect that plant miRNAs can completely reverse the developmental fate, i.e., turn worker into queen or queen into worker. It is worth noting that the inhibitory effects of plant miRNAs on honeybee development were gradually reduced from treatments with total pollen RNA to the miRNA pool and to only miR162a. For example, total pollen RNA prolonged the developmental time in honeybees and *Drosophila*, while miR162a did not. This phenomenon indicates that miR162a is not the sole active component, and other miRNAs, even larger RNAs, may also contribute to developmental regulation. Indeed, miR162a is only one of the multiple plant miRNAs enriched in beebread, and these miRNAs are only a portion of all classes of small RNAs, which themselves account for only a small fraction of total RNAs. Therefore, we propose that a single miRNA (i.e., miR162a) does not operate as an all-around regulator of caste development; instead, more plant RNA components likely function in a cooperative manner in the regulatory network leading to caste development. Similarly, the miR162a-*amTOR* pair is only one of the pathways that participate in this cross-kingdom regulation. The involvement of other regulatory pathways (e.g., those indicated in the bioinformatic analysis summarized in S7 Fig and S3 and S4 Tables) in honeybee development requires further investigation. In summary, the development of queens and workers is not determined by a single compound but, instead, is driven by the cooperation of multiple components in the larval food, which may include proteins, sugars, fatty acids and plant RNAs. However, why honeybees use such a sophisticated and intricate mechanism to regulate the queen-worker dimorphism is a fascinating question. For larvae that are destined to become queens, royal jelly is fed in copious amounts to drive the development of royal phenotypes. For worker-destined larvae, substantial quantities of plant miRNAs are absorbed when consuming beebread and pollen, thereby negatively influencing the larval development and inducing sterile worker bees. Reliance upon beebread and pollen as the exclusive food for sterile workers may have evolved in concert with the exploitation of plant miRNAs for caste regulation via a form of “RNAi castration”. The positive effects of royal jelly and the negative effects of beebread may maintain the stability of the colony’s social order and contribute to the survival of the colony in a coordinated manner. However, an opening question is raised regarding whether the plant miRNAs that reduce the development and fertility in honeybees and *Drosophila* have similar influences on solitary bees and bumblebees that would be exposed to the same plant miRNAs. Another opening question is about the widespread apicultural use of artificial pollen substitutes (commonly consist of protein sources derived from soy, wheat or lentils) in agricultural systems. Although the supplemental protein diets offset the poor nutritional conditions in honeybee colonies, long-term consumption of protein as the sole nutrition may compromise the ability of plant miRNAs to fine-tune honeybee development. Indeed, previous studies had explore the influence of natural pollen and artificial pollen substitutes on the cellular immunity, survival and parasite infection in honeybees and shown that the change from a natural to an artificial high nutritious diet in terms of protein content is not sufficient to promote healthy bees [61, 62]. If consumption of natural or artificial diets did produce varying levels of plant miRNAs in honeybees and impact the survival and breeding of honeybees deserves further investigation. Overall, our study uncovered a new layer of caste regulation in which plant RNAs are transmitted between species of different kingdoms, offering hints for understanding cross-kingdom interactions and co-evolution.

## Materials and methods

### Sample collection

The pollen used for this study was bee pollen, which are pollen pellets compressed and packed into corbicula on the outer surfaces of the hind legs after collection by forager bees. The pollen was separated using a specific collection device when bees come back to the comb. The royal jelly, honey, beebread and pollen were obtained in the cole or camellia flowering stage. All of the samples were stored at -80°C immediately after collection. Total RNA was extracted from royal jelly, honey, beebread and pollen using TRIzol Reagent (Invitrogen, Carlsbad, CA, USA). Small RNAs were extracted from royal jelly, honey, beebread and pollen using the MirVana Protein and RNA Isolation System (Ambion, Austin, TX, USA). Synthetic plant miRNA mimics and inhibitors and scrambled negative control RNAs were purchased from Invitrogen.

### Rearing of honeybee larvae under laboratory conditions

The diets (V.S. diet, D-1 diet and D-2 diet) for laboratory rearing of honeybee larvae have been described previously [22]. The V.S. diet for the first 3 days was as follows: 50% fresh royal jelly, 6% fructose, 6% glucose, 1% yeast extract and 37% dd-H_2_O. The D-1 diet for the next 2 days was as follows: 53% fresh royal jelly, 6% fructose, 6% glucose, 1% yeast extract and 34% dd-H_2_O. The D-2 diet for the following days and until pupation was as follows: 53% royal jelly, 7.8% fructose, 7.8% glucose, 1% yeast extract and 30.4% dd-H_2_O. A healthy colony was chosen for egg laying, and the queen was caged in an empty comb from 6:00–18:00. After 72 h, the hatched larvae were moved to 48-well plates, and total pollen RNA, small pollen RNAs, synthetic miRNA pool and synthetic miR162a were added to the diets. The detailed experimental procedure for preparation of the diets with added plant RNA (total pollen RNA, synthetic miRNA pool or synthetic miR162a) is shown in S12A Fig. DEPC-H_2_O was added to the diet as a control. The larvae were transferred to new plates with fresh diets every 12 h. The plates were kept in a crisper with 15.5% glycerine (90% relative humidity), and the crisper was placed in an incubator (33°C) during the larval period. Defecating larvae were transferred into new 24-well plates, and each well contained a piece of filter paper. The plates containing defecating larvae were kept in a crisper with a saturated sodium chloride solution (70% relative humidity), and the crisper was left in an incubator (33°C). Then, the newly emerged adults were collected, and their characteristics were measured. At the beginning, we moved 48 larvae into the plates for each group and generally got 25–30 emerged adults due to the mortality during *in vitro* rearing. The honeybee larvae cultivated in this laboratory conditions largely developed to intermediates with characteristics between a worker and queen. For example, they generally had ovarioles (30–80 ovarioles) more than natural worker bees (< 10 ovarioles) but less than queens (> 150 ovarioles).

### Rearing of *Drosophila*

A total of 20–30 pairs of *Drosophila* were caged in a tube containing ~15 mL of medium from 10:00–16:00 for egg laying (10–15 tubes for each experimental group). Total pollen RNA, small pollen RNAs, the synthetic miRNA pool and synthetic miR162a were added to the medium. The detailed experimental procedure for preparation of the medium with added plant RNA (total pollen RNA, small pollen RNAs, synthetic miRNA pool or synthetic miR162a) is shown in S12B Fig. DEPC-H_2_O was added to the medium as a control. Approximately 8–9 days later, newly enclosed adults were collected, and their characteristics were measured. We generally got 25–35 enclosed adults at this stage. On day 5 after eclosion, 5 pairs of *Drosophila* were placed in a custom tube for fertility analyses (10–15 tubes for each experimental group). The eggs that were laid by the 5 pairs of *Drosophila* were counted every day for 5 days. The culture environments for each parallel test were carefully controlled, and we only compared results obtained in the same parallel test, which excludes confounding environmental factors that may otherwise affect experimental results.

### Illumina deep-sequencing

The sequencing procedure was conducted as previously described [15]. Briefly, fresh samples of royal jelly, honey, beebread and pollen were collected from colonies of Italian honeybees. Total RNA was extracted from 10 g of these samples using Trizol Reagent (Invitrogen, Carlsbad, CA, USA) according to the manufacturer’s instructions. Then, equal amounts of total RNA were analysed using Illumina deep-sequencing technology, and the sequencing procedure was performed by BGI (Shenzhen, China). After masking the adaptor sequences from the raw data and removing short and low-quality reads, a total of 9,548,986, 13,683,503, 9,559,836 and 9,561,153 reads from royal jelly, honey, beebread and pollen of cole and 8,996,733, 12,160,200, 15,237,283 and 16,690,115 reads from royal jelly, honey, beebread and pollen of camellia were obtained, respectively. The clean reads were aligned to the transcript sequences using bowtie 1.1.2 (http://bowtie-bio.sourceforge.net) with perfect match. Transcript sequences of *Apis mellifera* (assembly Amel_4.5) and *Brassica napus* (assembly Brassica_napus_assembly_1.0) were downloaded from the NCBI genome database (https://www.ncbi.nlm.nih.gov/genome). Clean reads were also compared to the known miRNA precursors in the miRBase database 21.0 based on the Smith-Waterman algorithm. Only candidates with no mismatches and no more than 2 shifts were counted as miRNA matches. For normalization, the total sequencing frequency of each sample was normalized to 10,000,000. Data for Illumina deep-sequencing have been deposited at GEO with the accession code GSE76286 (http://www.ncbi.nlm.nih.gov/geo/query/acc.cgi?token=yrevmigijrynjsl&acc=GSE76286).

### qRT-PCR

To determine the plant miRNA levels in honeybee larval food, total RNA was extracted from royal jelly, honey, beebread and pollen using Trizol Reagent (Invitrogen) according to the manufacturer’s instructions. To determine the *amTOR*, *dmTOR* and miR162a levels in honeybees or *Drosophila*, newly emergence adults were collected, and total RNA was extracted using Trizol Reagent (Invitrogen).

Assays to quantify mature miRNAs were performed using TaqMan miRNA probes (Applied Biosystems, Foster City, CA) according to the manufacturer’s instructions. Briefly, 1 μg of total RNA was reverse-transcribed to cDNA using AMV reverse transcriptase (TaKaRa, Dalian, China) and a stem-loop RT primer (Applied Biosystems). The following reaction conditions were used: 16°C for 30 min, 42°C for 30 min, and 85°C for 5 min. Real-time PCR was performed using a TaqMan PCR kit on an Applied Biosystems 7500 Sequence Detection System (Applied Biosystems). The reactions were incubated in a 96-well optical plate at 95°C for 5 min, followed by 40 cycles of 95°C for 15 sec and 60°C for 1 min. All of the reactions were run in triplicate. After the reactions, cycle threshold (C_T_) values were determined using fixed threshold settings, and the mean C_T_ of triplicate PCRs was determined. To calculate the absolute expression levels of the target miRNAs, a series of synthetic miRNA oligonucleotides at known concentrations were reverse transcribed and amplified. The absolute amount of each miRNA was then calculated in reference to the standard curve. For cross-sample comparisons of miRNAs in royal jelly, honey, beebread and pollen, miRNA levels were normalized to the total amounts of RNA or to the total mass of the samples.

To quantify *amTOR* and *dmTOR* mRNA, 1 μg of total RNA was reverse-transcribed to cDNA using a specific reverse primer and AMV reverse transcriptase (TaKaRa) under the following conditions: 16°C for 15 min, 42°C for 60 min, and 85°C for 5 min. Subsequently, real-time PCR was performed using the RT product, SYBR Premix Ex Taq (Takara, Dalian, China) and specific primers for *amTOR* and *dmTOR*. The primers that were used in this study were as follows: *amTOR*-forward, 5’-TTGGTTGGGTACCGCATTGT-3’; *amTOR*-reverse, 5’-AACCTGGGGCCATTCTTAGC-3’; *dmTOR*-forward, 5’-CTCTTACATGAATCCGATCCTCA-3’; and *dmTOR*-reverse, 5’-CGGAGCCTCCATTAACCT-3’. The reactions were incubated at 95°C for 5 min, followed by 40 cycles at 95°C for 15 sec, 55°C for 30 sec, and 72°C for 30 sec. After the reactions were complete, C_T_ values were determined using fixed threshold settings. The relative amounts of *amTOR* and *dmTOR* were normalized to *amActin* and *dmActin*, respectively. The primers for *amActin* and *dmActin* were as follows: *amActin*-forward, 5’-TGCCAACACTGTCCTTTCTG-3’; *amActin*-reverse, 5’-AGAATTGACCCACCAATCCA-3’; *dmActin*-forward, 5’-CGCGATTTGACCGACTACCT-3’; and *dmActin*-reverse 5’-TTGATGTCACGGACGATTTCA-3’.

### Northern blotting analysis

Small RNAs were extracted from royal jelly, honey, beebread and pollen using the MirVana Protein and RNA Isolation System (Ambion, Austin, TX, USA). The northern blot analysis was carried out using miRCURY LNA microRNA Detection Probes with DIG-labelling (Exiqon, Woburn, MA, USA) and a DIG luminescence detection kit (Roche, Indianapolis, IN, USA) according to the manufacturer’s instructions. Briefly, samples of small RNAs (15 μg) and synthesized size markers (Invitrogen) were added to Gel Loading Buffer II (Ambion) and denatured at 95°C for 5 min. A 15% TBE-urea gel was pre-run at 250 V for 60 min, and the samples and size markers were added to the gel and run at 250 V until the bromophenol blue (BPB) from the loading solution reached approximately 1 cm above the bottom of the gel. Generally, BPB and cyanol from the loading solution run at approximately 15 bases and 60 bases, respectively. RNA was then transferred onto a nylon membrane (Hybond N+, Amersham Biosciences) via electroblotting at 250 mA in 0.5× TBE (Tris-borate-EDTA) buffer for 1 h. After UV-crosslinking at 1200 mJ, a prehybridization step was performed by incubating the membrane with 40 mL of ULTRAhyb-Oligo solution (Ambion) pre-heated to 50°C. Prehybridization was performed for 30 min at 50°C in a standard rotating hybridization oven. DIG-labelled LNA probes were hybridized to the membranes overnight at 50°C with slow rotation. The next day, the membrane was washed twice for 15 min each in NorthernMax Low-Stringency wash solution no. 1 (Ambion) at 50°C, briefly rinsed for 10 min with Washing Buffer from the DIG wash and Block Buffer Set (Roche), blocked for 30 min in 1× Blocking Solution (Roche), incubated for 30 min in antibody solution (anti-DIG-AP 1:10,000 in 1× Blocking solution, Roche), washed twice for 15 min each with Washing Buffer and incubated for 2–5 min with 1× Detection Buffer (Roche). Then, the membrane was incubated with CSPD, the chemiluminescent substrate for alkaline phosphatase (Roche) and exposed to Amersham Hyperfilm ECL (GE Healthcare Life Sciences, Piscataway, NJ) following the instructions of the DIG Luminescent Detection Kit (Roche).

### Target prediction and GO analysis

Sequence information of honeybee mRNAs was collected from the NCBI database. Two bioinformatic algorithms, RNAhybrid and miRanda [63, 64], were used in combination to scan honeybee mRNAs for potential binding sites for plant miRNAs. The gene lists generated by miRNA target prediction were assigned to orthology groups with *Drosophila melanogaster* genes on the basis of BLAST match, and GO terms were assigned to bee genes based on annotation of *Drosophila* genes. GO functional terms and *Drosophila* gene GO annotations were downloaded from the GO database. Counts of genes in specific categories were performed by using PANTHER, a gene functional classification tool. χ2 tests were performed in R, and differences were considered statistically significant at p < 0.05. Cytoscape was used to build the GO network associations.

### Cell line, plasmid construction and transfection

We utilized the processing machinery of pri-dme-mir-184 to express miR162 in *Drosophila* S2 cells. The S2 cell line was cultured at 28°C with Schneider’s *Drosophila* medium containing 10% heat-inactivated FBS. The miR162a sequence was substituted into a 300-bp pri-dme-mir-184 backbone with structurally conserved nucleotide changes to maintain pairing. The 300-bp pri-dme-mir-184 was GTTTTCTATTCACGCTTTAGTGCACTTATTTACTCGATTGTATGATCCAAAGCTCCTCTTTGACTCGCCGAATTCCTGTCGATTCAATGGGTATTGGTTTGGTTGGCCGGTGCATTCGTACCCTTATCATTCTCTCGCCCCGTGTGCACTTAAAGACAACTGGACGGAGAACTGATAAGGGCTCGTATCACCAATTCATCCTCGGGTCAGCCCAGTTAATCCACTGATTTGCACACTTTTCTTTATACATACGAGGATACTTACCCCACGTTTCGATTACGCGCATCAATCAATCAATCA, and the underlined parts were replaced with TCGATAAACCTCTGCATCCAG and AATGAATGAGAGGCTTTATCGA, respectively. The 300-bp fragment containing the miR162a sequence was synthesized directly and cloned into a pAc5.1 vector. Cultured cells were prepared for transfection by seeding 1×106 cells/mL in a 24-well plate. After culturing the cells for 12–18 h, transfection was performed with Effectene transfection reagent (Qiagen, Valencia, CA, USA). The transfection mixture per well contained 6 μL of Effectene reagent only, 6 μL of Effectene reagent and 0.3 μg of miR162a expressing plasmids, or 6 μL of Effectene reagent and 0.3 μg of pAc5.1 vectors without any insert. The cells were collected 48 h after transfection and used for western blotting analysis.

### Western blotting analysis

Plasmids expressing miR162a were transfected into S2 cells using Effectene (Qiagen) according to the manufacturer’s instructions. The cells were lysed in RIPA buffer (0.5% NP-40, 0.1% sodium deoxycholate, 150 mM NaCl, 50 mM Tris-HCl (pH 7.5)). The lysates were resolved via 6% SDS-PAGE (for the dmTOR protein) or 10% SDS-PAGE (for internal control GAPDH protein), transferred to a PVDF membrane (Millipore, Bedford, MA, USA) and probed with anti-dmTOR or anti-GAPDH antibodies (Santa Cruz Biotechnology, CA, USA). Anti-dmTOR antibodies were polyclonal antibodies that were custom-made by GenScript USA Inc. (Nanjing, China). The epitope was predicted using the GenScript OptimumAntigen design tool, and the peptide antigen was then synthesized. After the coupling reaction and mixing with complete adjuvant, the coupled antigen was used once for a subcutaneous injection. The host strain was a New Zealand rabbit. Then, the coupled antigen was mixed with incomplete adjuvant and injected into the rabbit. Subsequently, serum was taken from the immunized rabbit, and the antibody was purified.

### Pull-down assay

A DNA probe complementary to *dmTOR* was synthesized with 5’ and 3’ terminal biotin labels. The probe was dissolved in a wash/binding buffer (0.5 M NaCl; 20 mM Tris-HCl, pH 7.5; 1 mM EDTA) to a concentration of 8 pmol/μL. Then, the probe was incubated with streptavidin magnetic beads (New England Biolabs) at room temperature for 1 h with occasional agitation. After incubation, the probe-coated beads were washed twice and captured with a magnet to remove the supernatant. The total RNA that was extracted from miR162a-transfected S2 cells (50~100 μg) was pretreated with DNaseI and then heated at 65°C for 5 min, followed immediately by an ice bath. Then, the RNA was incubated with the prepared probe-coated beads at 37°C for 3 h with occasional agitation, and the beads were washed twice with wash/binding buffer and once with a cold low-salt buffer (0.15 M NaCl; 20 mM Tris-HCl, pH 7.5; 1 mM EDTA). After each wash, a magnet was applied to the tube, and the supernatant was removed. Finally, the RNA was eluted from the probe-coated streptavidin beads with Elution Buffer (10 mM Tris-HCl, pH 7.5; 1 mM EDTA) prewarmed to 90°C and then analysed via qRT-PCR. The following probe sequences were used: anti-*dmTOR* pull-down probe 5’-CTAGAGCCCAAGTCTGCATTGAA-3’ and random pull-down probe 5’-GGCAGCTAACCTATATGACATGC-3’.

### *Drosophila* genetics, immunohistochemistry and microscopy

*Drosophila* were cultured following standard procedures at 25°C except for the transgenic lines, which were cultured at 29°C. Strain w^1118^ was obtained from the Bloomington *Drosophila* Stock Center. To generate the transgenic line, the miR162a binding sequence in the *dmTOR* gene was cloned into a pUbi-GFP expression vector, and the pUbi-GFP-*dmTOR* transgenic line was obtained via embryo injection according to standard procedures. After miR162a or mutant miR162a was added to the larval diets of the transgenic *Drosophila*, the ovaries of transgenic *Drosophila* were dissected in PBS and then fixed in a devitellinizing buffer (100 μl, 7% formaldehyde) and heptane (600 μl) mixture for 10 minutes. After 3 washes in PBS for 10 min each, ovaries were incubated in blocking solution (PBT, 10% goat serum) for 30 min. GFP levels were observed and compared between different groups.

### Statistical analysis

The analyses were performed using IBM SPSS Statistics 19. One-way ANOVAs and two-tailed Student’s *t*-tests were used for the analyses. The data are presented as the means ± SEM of at least three independent experiments, and differences were considered statistically significant at p < 0.05.

## Supporting information

S1 Fig The distribution of small RNAs of various lengths (9–33 bp). RNA was extracted from royal jelly, honey, beebread and pollen collected during the cole (*Brassica campestris*) flowering stage and analysed using Illumina deep-sequencing technology.
(TIF)

S2 Fig Comparison of the levels of plant and animal miRNAs in royal jelly, honey, beebread and pollen. (**a**) Comparison of the levels (sequencing reads) of plant and animal miRNAs between royal jelly and beebread and between pollen and beebread collected during the cole (*Brassica campestris*) flowering stage. (**b**) Comparison of the levels (sequencing reads) of plant and animal miRNAs between royal jelly and beebread and between pollen and beebread collected during the camellia (*Camellia japonica*) flowering stage. (**c**) The absolute levels of 16 representative plant miRNAs in royal jelly and beebread as detected via qRT-PCR. miRNA levels were normalized to the total amounts of RNA or the mass of the samples. Data are represented as the mean ± SEM.
(TIF)

S3 Fig Increase in representative plant miRNAs in the diets that were supplemented with pollen RNA. To make the beebread mimic, total pollen RNA was purified from 1 g of pollen and added to 10 g of larval diet. Plant miRNAs were generally present in beebread mimic in a similar range of concentration (same order of magnitude) as those in beebread.
(TIF)

S4 Fig Effects of plant RNA on the development and survival of honeybee larvae. (**a**) Representative images showing the body size of 3.5-day-old larvae reared with control diets or 0.5-, 1- or 2-fold of beebread mimic. (**b**) Survival rate of developing larvae reared with control diets or 0.5-, 1- or 2-fold of beebread mimic.
(TIF)

S5 Fig Increase in representative plant miRNAs in honeybees and *Drosophila* that received a diet containing plant miRNAs. (**a-c**) qRT-PCR analysis of the levels of 16 representative plant miRNAs or miR162a in 4^th^ instar honeybee larvae reared with the control diet or diets supplemented with total pollen RNA (a), the synthetic miRNA pool (b) or synthetic miR162a (c). (**d-f**) qRT-PCR analysis of the levels of 16 representative plant miRNAs or miR162a in 3-day-old *Drosophila* larvae reared with control medium or medium supplemented with total pollen RNA (d), the synthetic miRNA pool (e) or synthetic miR162a (f).
(TIF)

S6 Fig Representative images showing the body size of 3.5-day-old larvae (a) and newly emerged adults (b) reared with the control diet or beebread mimic.
(TIF)

S7 Fig Enrichment of genes belonging to common functional categories. A list of orthologous genes of honeybee and Drosophila was examined for significant associations with specific GO functional categories based on the annotation of Drosophila genes. The circle size and color represents the p-value of GO terms. Genes in large and red circles have relatively lower p-values than genes in small and yellow circles.
(TIF)

S8 Fig Identification of plant miRNAs that can target *amTOR* via luciferase reporter screening. Firefly luciferase reporters containing the potential binding sites for plant miRNAs in the *amTOR* gene were co-transfected with scrambled RNA or plant miRNAs into 293T cells. At 24 h post-transfection, cells were assayed using luciferase assay kits. Data are represented as the mean ± SEM. **p < 0.01, Student’s *t*-test.
(TIF)

S9 Fig Decrease of *amTOR* mRNA in honeybees and *dmTOR* mRNA in *Drosophila* that received a diet containing plant miRNAs. (**a-b**) qRT-PCR analysis of the levels of *amTOR* mRNA in 4^th^ instar honeybee larvae reared with control diets or diets supplemented with total pollen RNA (a) or the synthetic miRNA pool (b). Error bars represent SEM. *p < 0.05; ***p < 0.001, Student’s t test or one-way ANOVA. (**c-d**) qRT-PCR analysis of the levels of *dmTOR* mRNA in 3-day-old *Drosophila* larvae reared with control medium or medium supplemented with total pollen RNA (c) or the synthetic miRNA pool (d). Error bars represent SEM. ***p < 0.001, Student’s *t* test.
(TIF)

S10 Fig Exclusion of the possibility that the potential remaining chemical regents left after RNA isolation caused the observed phenotypic changes in *Drosophila*. H_2_O instead of pollen was processed for RNA isolation and added to the diet as a mock control. Developmental time (a), body weight (b) and ovary size (c) of *Drosophila* that were reared with control medium or mock medium (n = 25–30). Error bars represent SEM. Student’s *t* test.
(TIF)

S11 Fig Effects of small plant RNA on *Drosophila* phenotypes. Developmental time (a), body weight (b), body length (c), ovary size (d) and fecundity (e) of *Drosophila* reared with control medium or medium containing small pollen RNA (n = 25–35). Error bars represent SEM. *p < 0.01; **p < 0.05; ***p < 0.001, Student’s *t* test.
(TIF)

S12 Fig The detailed experimental procedure for preparation of the diets for honeybee (a) and *Drosophila* (b).
(TIF)

S1 Table Data for Illumina deep-sequencing of royal jelly, honey, pollen and beebread collected during *Brassica campestris* flowering stage. (PDF)

S2 Table Data for Illumina deep-sequencing of royal jelly, honey, pollen and beebread collected during *Camellia japonica* flowering stage. (PDF)

S3 Table Functional analysis of gene lists using GO terms. A list of orthologous genes of honeybee and Drosophila was examined for significant associations with specific GO functional categories based on the annotation of Drosophila genes. Among the 10 top-ranked GO categories, 6 are directly related to development process. As a control, none of the irrelevant GO process is related to development process.
(PDF)

S4 Table Potential target genes for individual plant miRNAs. (PDF)
