# Supplementary figures and images for: Plant microRNAs in larval food regulate honeybee caste development
Source: PLoS Genet. 2017 Aug 31;13(8):e1006946. doi: 10.1371/journal.pgen.1006946 (PMC5578494; doi:10.1371/journal.pgen.1006946)

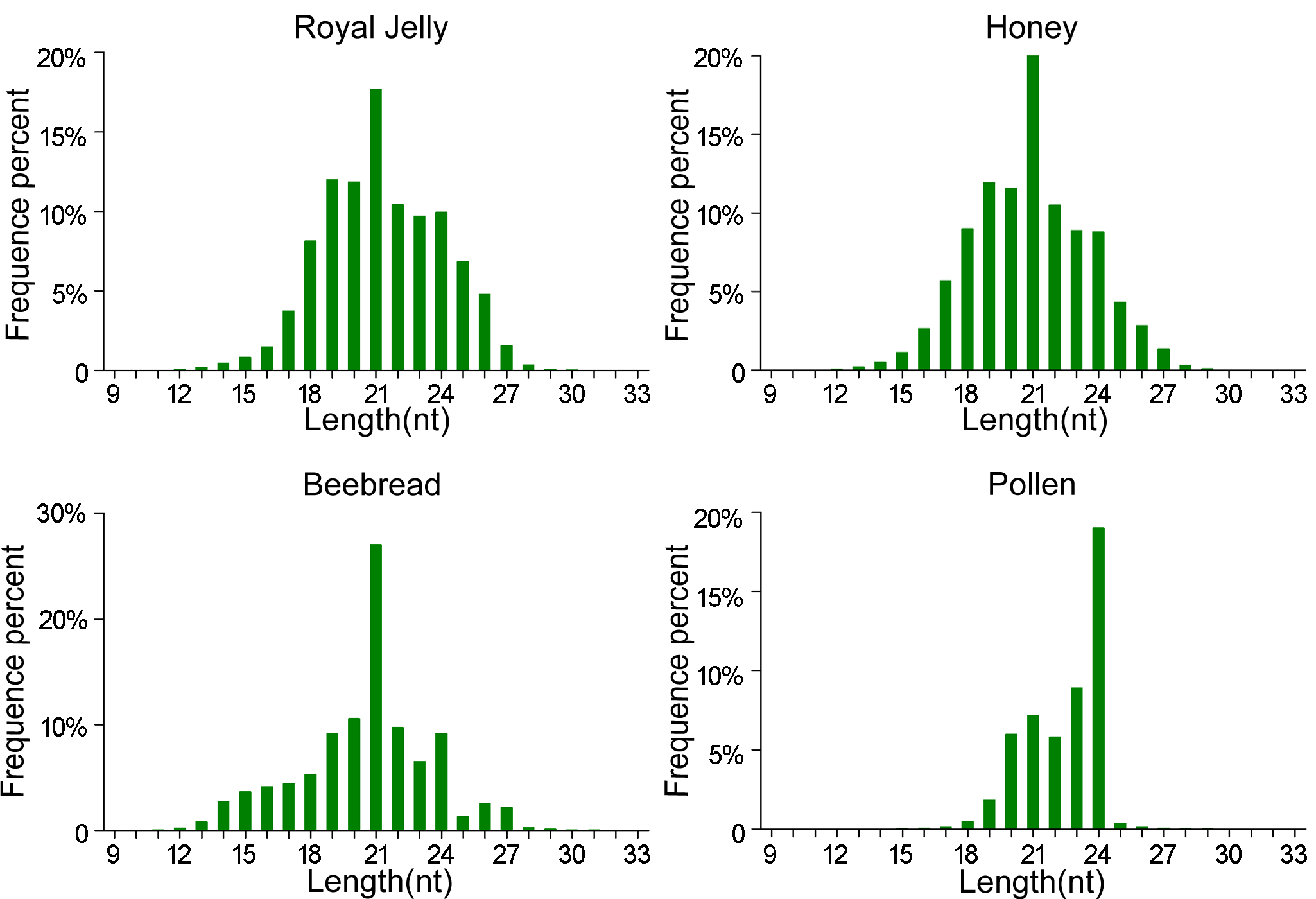

Supplement: S1 Fig — RNA was extracted from royal jelly, honey, beebread and pollen collected during the cole (Brassica campestris) flowering stage and analysed using Illumina deep-sequencing technology. (TIF) [file pgen.1006946.s001.tif]

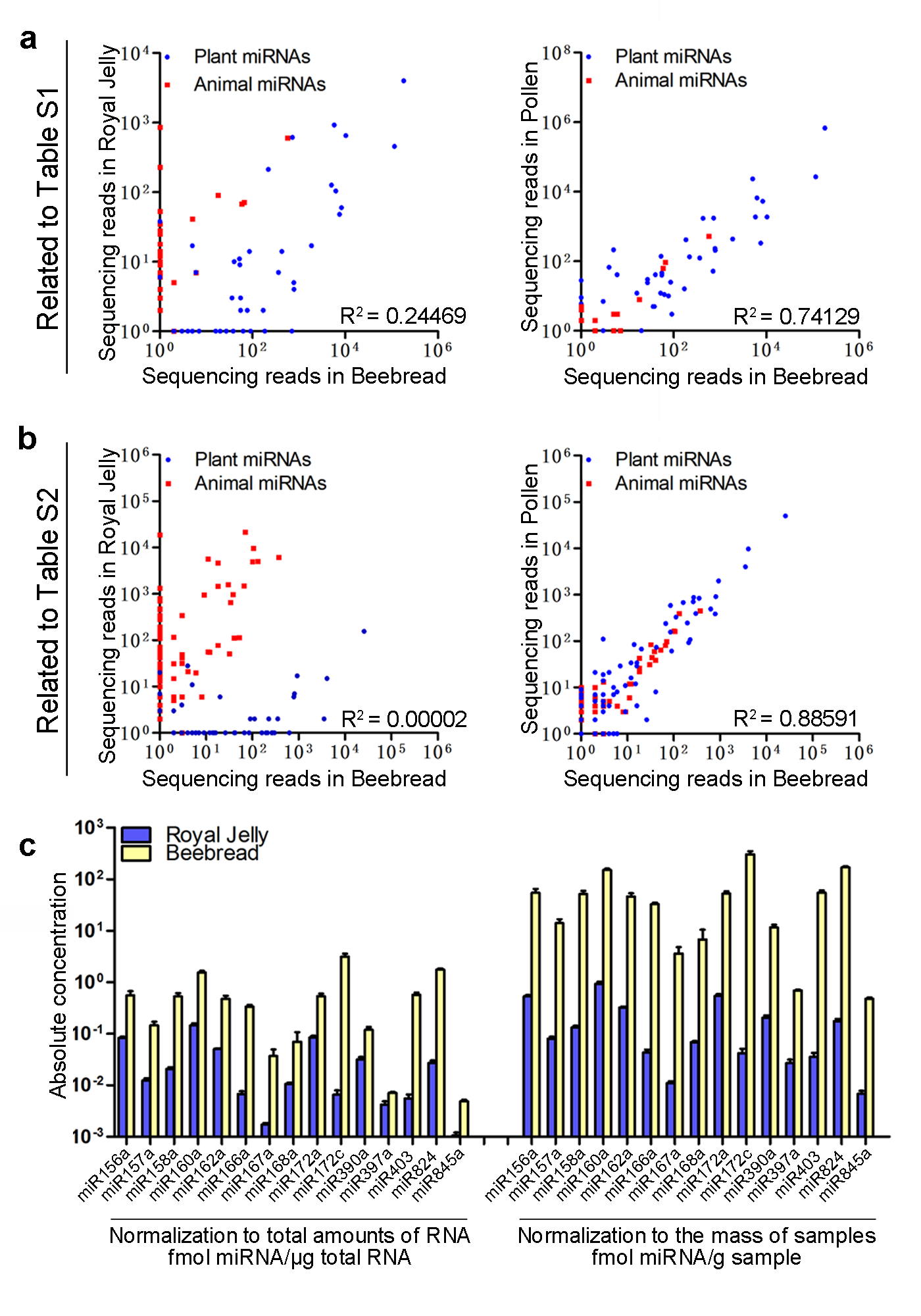

Supplement: S2 Fig — (a) Comparison of the levels (sequencing reads) of plant and animal miRNAs between royal jelly and beebread and between pollen and beebread collected during the cole (Brassica campestris) flowering stage. (b) Comparison of the levels (sequencing reads) of plant and animal miRNAs between royal jelly and beebread and between pollen and beebread collected during the camellia (Camellia japonica) flowering stage. (c) The absolute levels of 16 representative plant miRNAs in royal jelly and beebread as detected via qRT-PCR. miRNA levels were normalized to the total amounts of RNA or the mass of the samples. Data are represented as the mean ± SEM. (TIF) [file pgen.1006946.s002.tif]

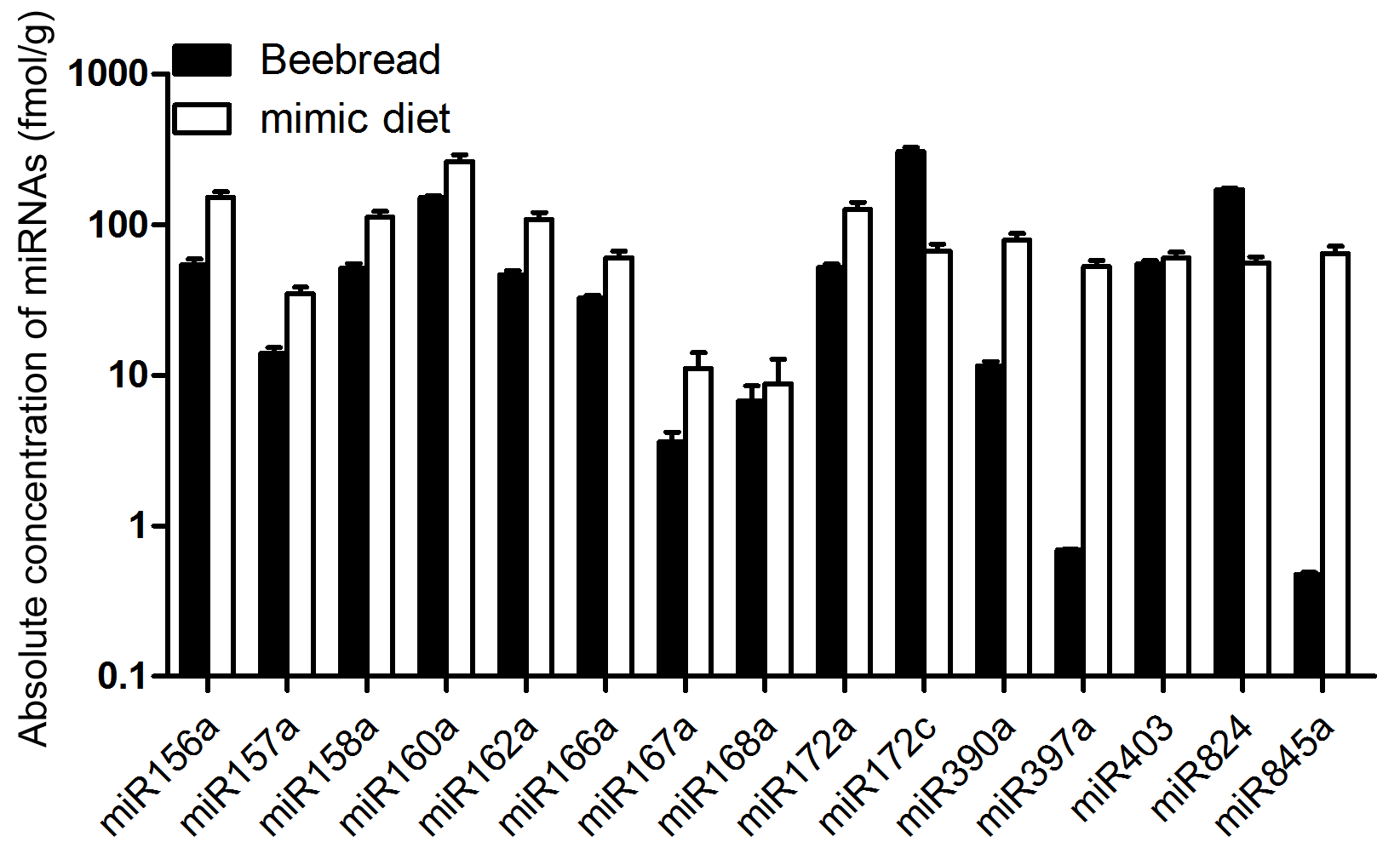

Supplement: S3 Fig — To make the beebread mimic, total pollen RNA was purified from 1 g of pollen and added to 10 g of larval diet. Plant miRNAs were generally present in beebread mimic in a similar range of concentration (same order of magnitude) as those in beebread. (TIF) [file pgen.1006946.s003.tif]

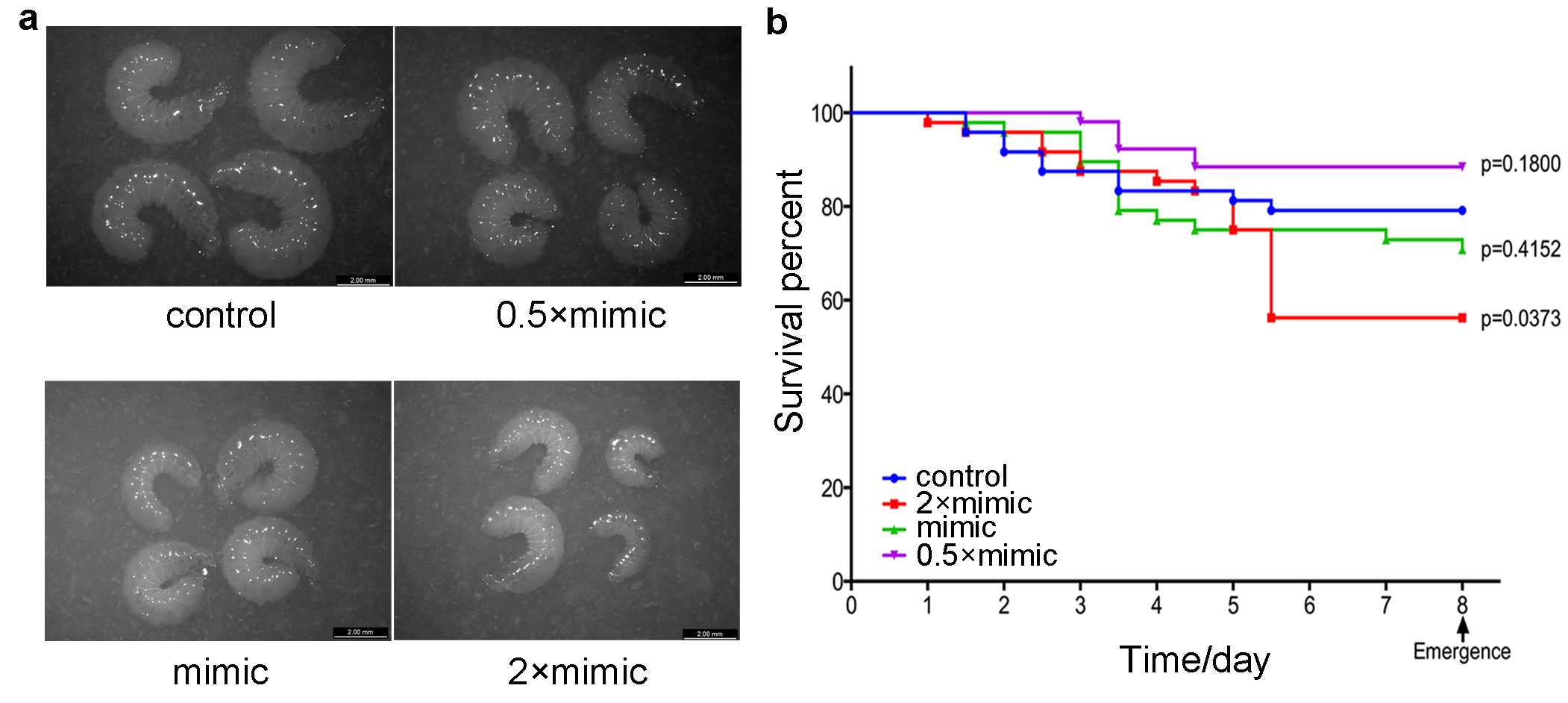

Supplement: S4 Fig — (a) Representative images showing the body size of 3.5-day-old larvae reared with control diets or 0.5-, 1- or 2-fold of beebread mimic. (b) Survival rate of developing larvae reared with control diets or 0.5-, 1- or 2-fold of beebread mimic. (TIF) [file pgen.1006946.s004.tif]

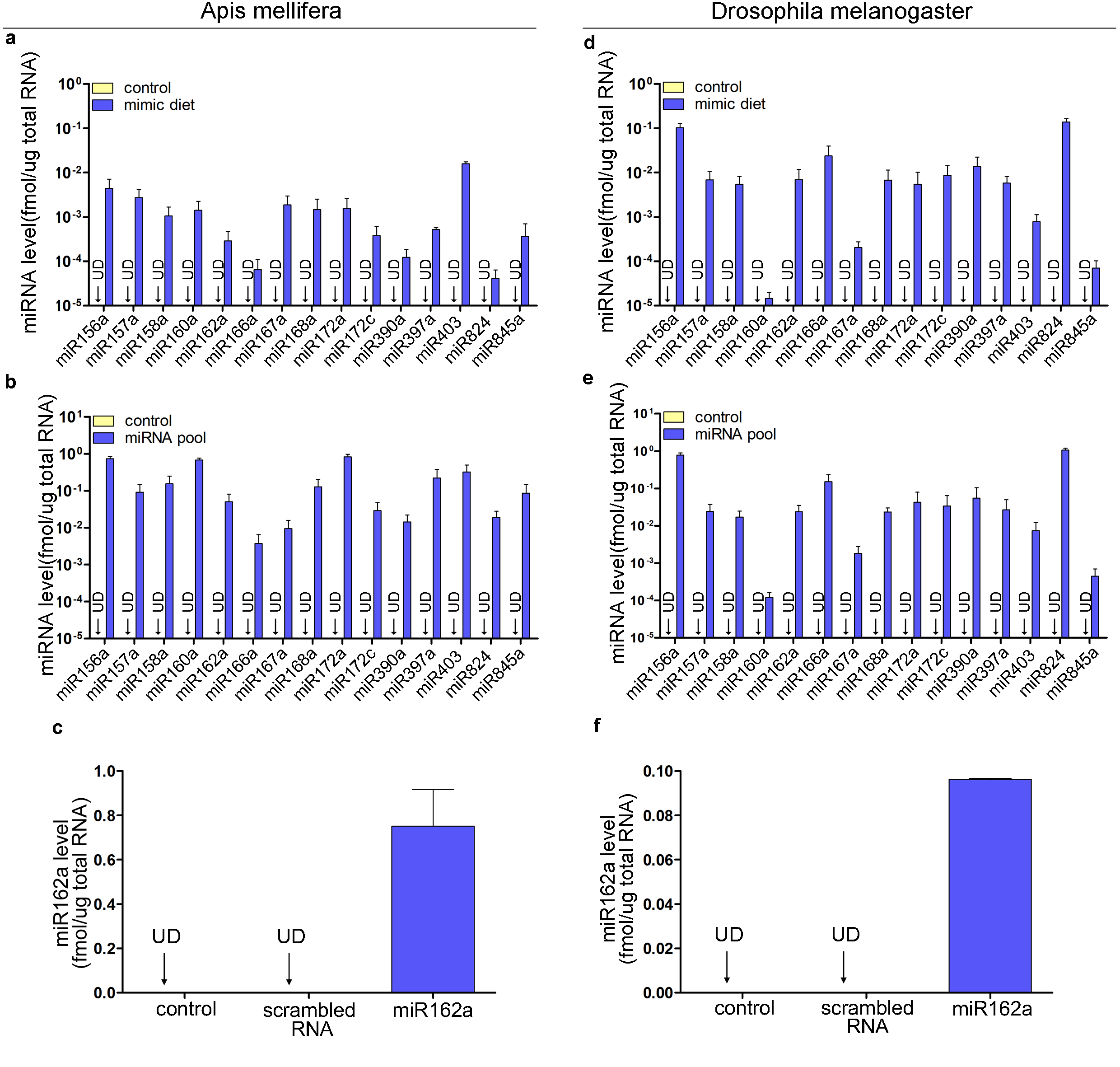

Supplement: S5 Fig — (a-c) qRT-PCR analysis of the levels of 16 representative plant miRNAs or miR162a in 4th instar honeybee larvae reared with the control diet or diets supplemented with total pollen RNA (a), the synthetic miRNA pool (b) or synthetic miR162a (c). (d-f) qRT-PCR analysis of the levels of 16 representative plant miRNAs or miR162a in 3-day-old Drosophila larvae reared with control medium or medium supplemented with total pollen RNA (d), the synthetic miRNA pool (e) or synthetic miR162a (f). (TIF) [file pgen.1006946.s005.tif]

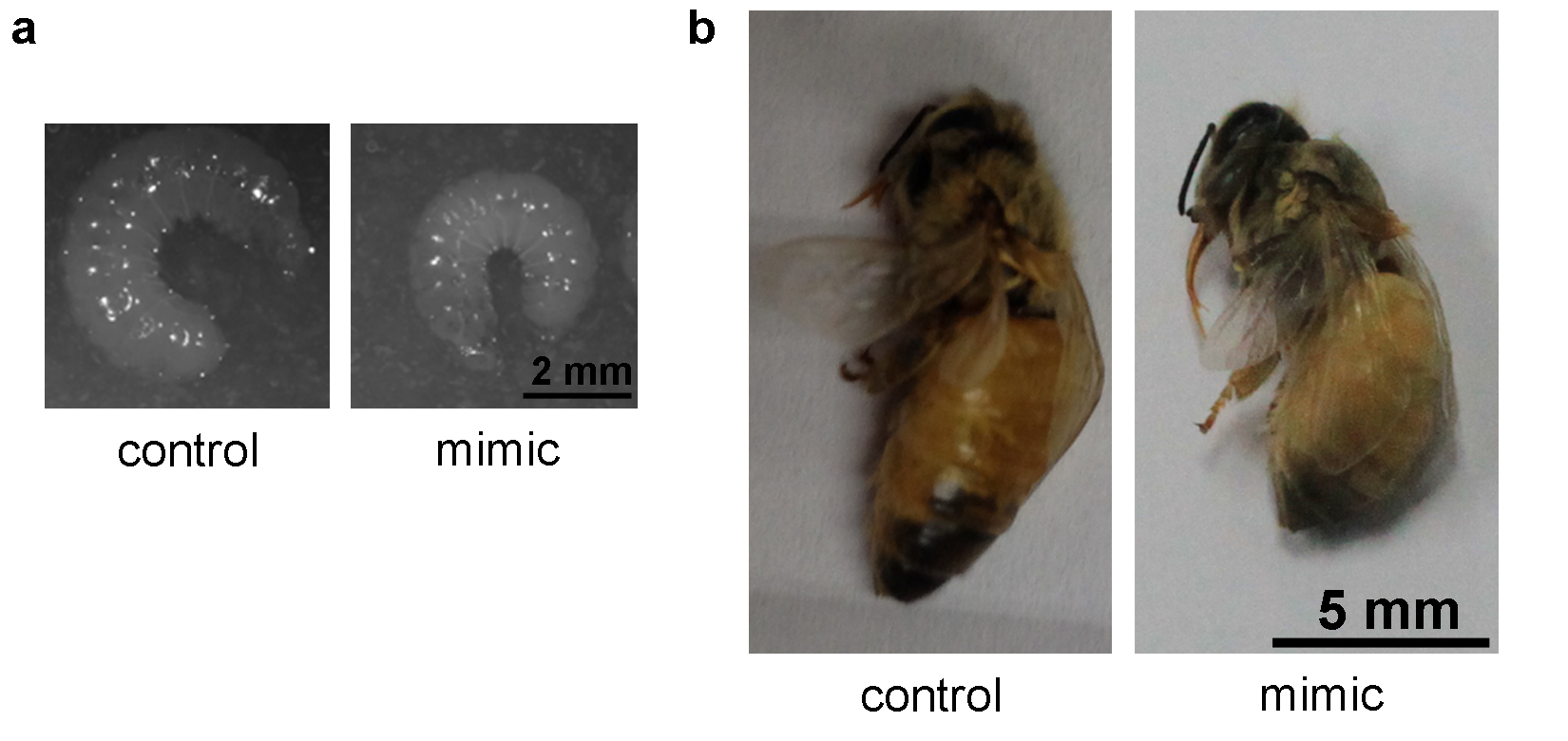

Supplement: S6 Fig — Representative images showing the body size of 3.5-day-old larvae (a) and newly emerged adults (b) reared with the control diet or beebread mimic. (TIF) [file pgen.1006946.s006.tif]

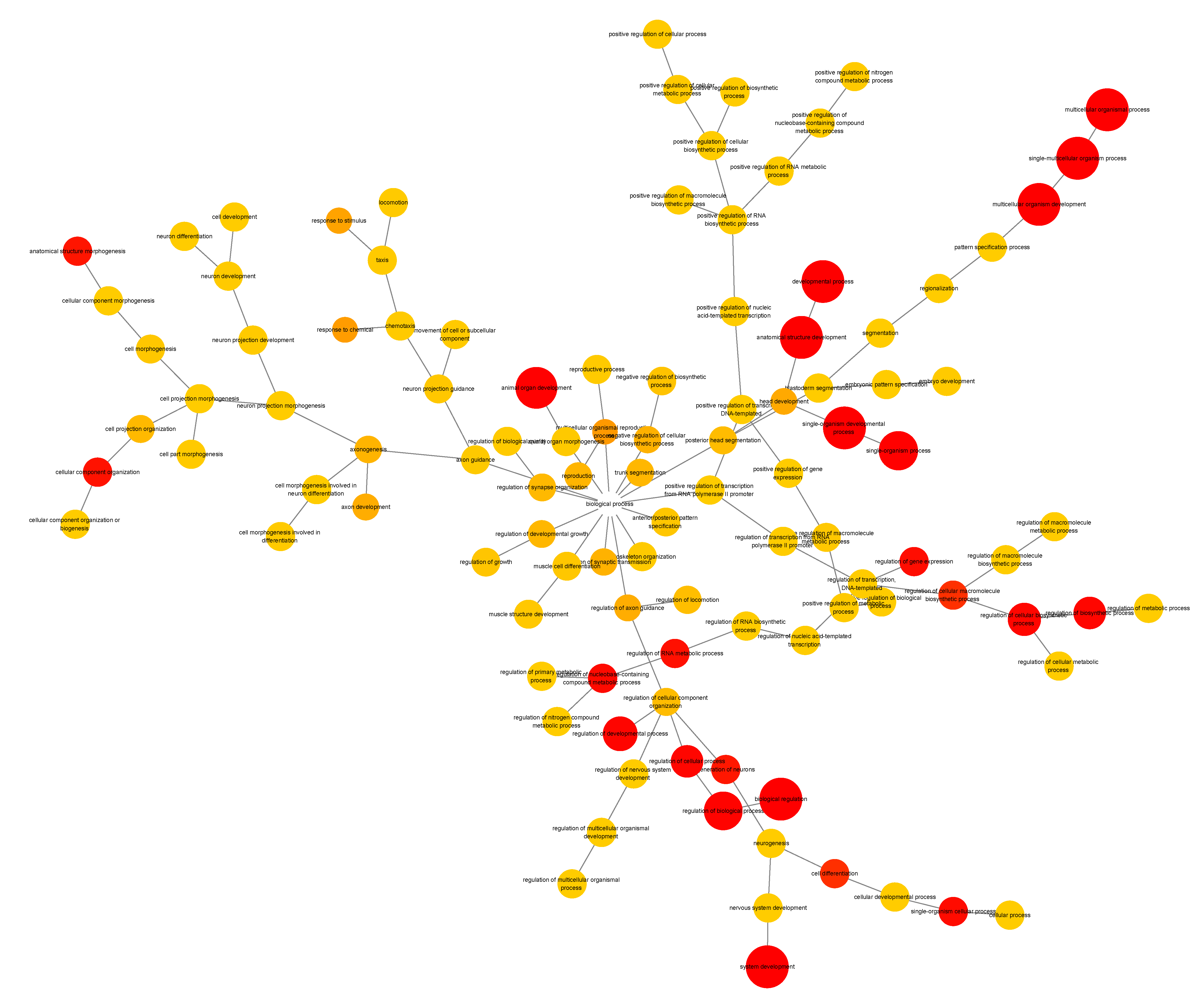

Supplement: S7 Fig — A list of orthologous genes of honeybee and Drosophila was examined for significant associations with specific GO functional categories based on the annotation of Drosophila genes. The circle size and color represents the p-value of GO terms. Genes in large and red circles have relatively lower p-values than genes in small and yellow circles. (TIF) [file pgen.1006946.s007.tif]

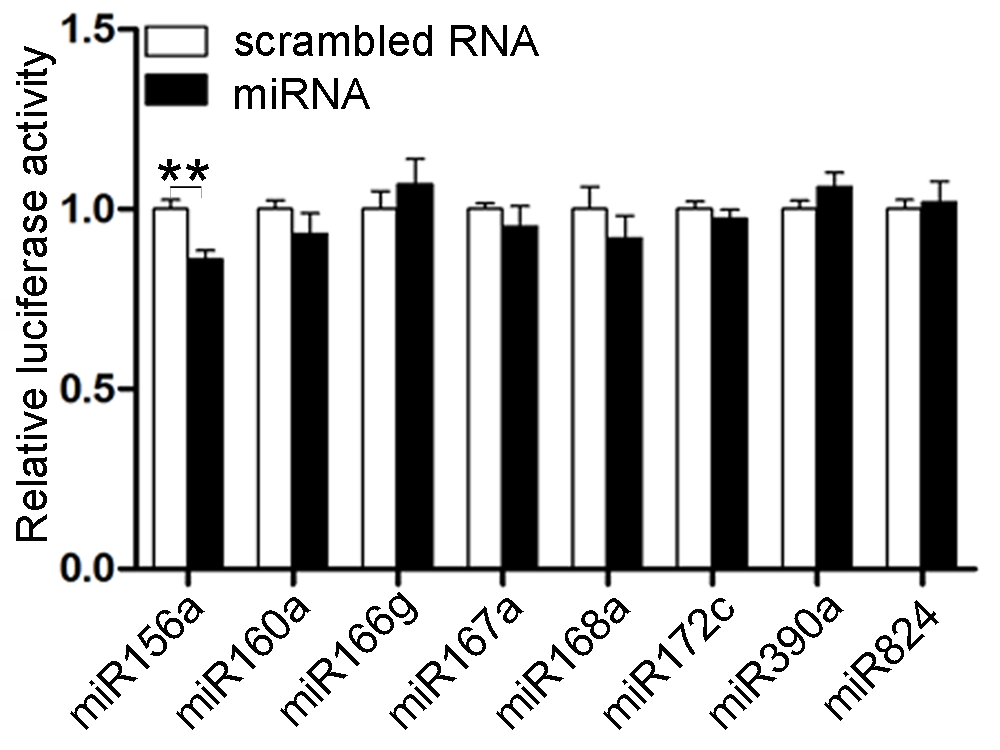

Supplement: S8 Fig — Firefly luciferase reporters containing the potential binding sites for plant miRNAs in the amTOR gene were co-transfected with scrambled RNA or plant miRNAs into 293T cells. At 24 h post-transfection, cells were assayed using luciferase assay kits. Data are represented as the mean ± SEM. **p < 0.01, Student’s t-test. (TIF) [file pgen.1006946.s008.tif]

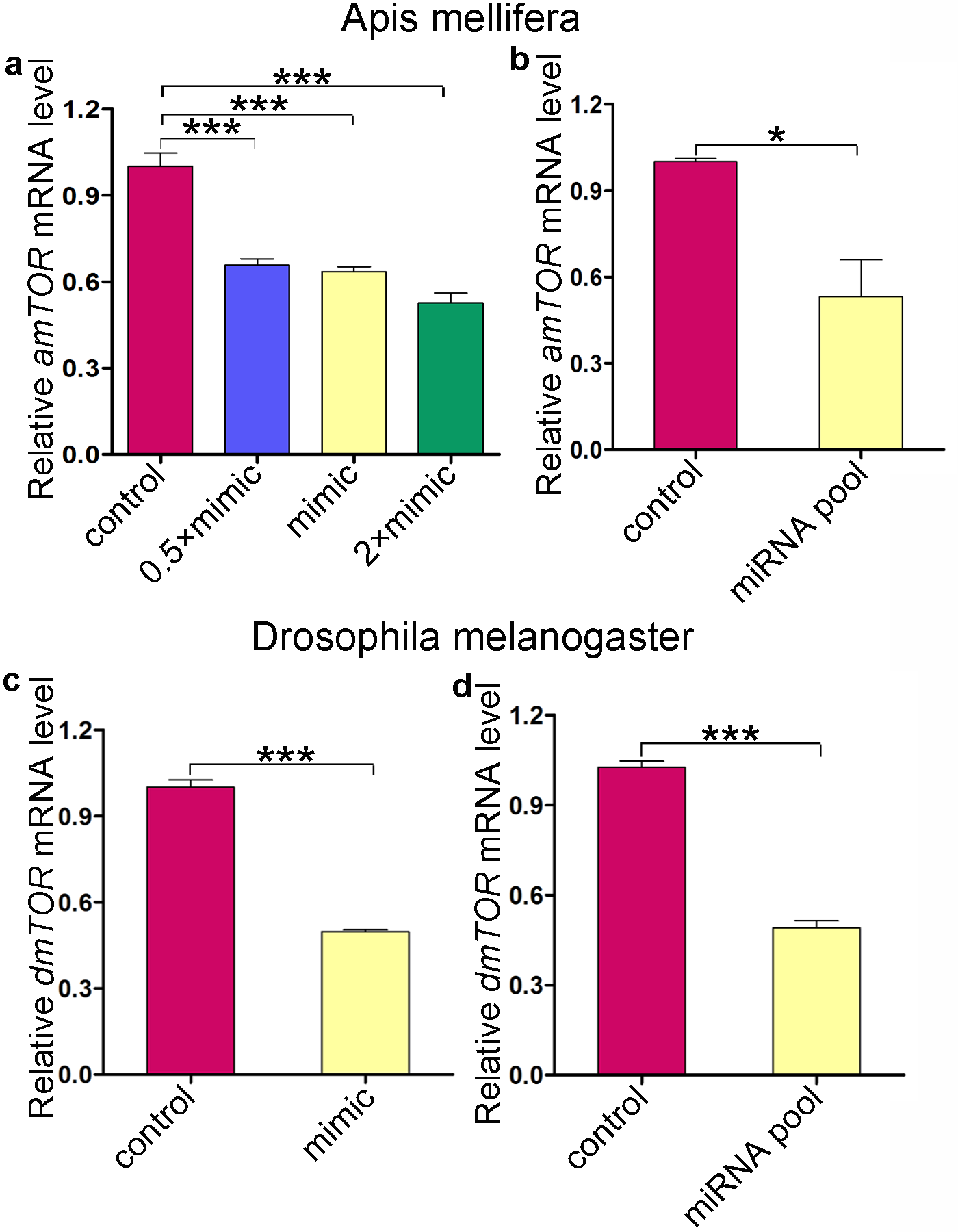

Supplement: S9 Fig — (a-b) qRT-PCR analysis of the levels of amTOR mRNA in 4th instar honeybee larvae reared with control diets or diets supplemented with total pollen RNA (a) or the synthetic miRNA pool (b). Error bars represent SEM. *p < 0.05; ***p < 0.001, Student’s t test or one-way ANOVA. (c-d) qRT-PCR analysis of the levels of dmTOR mRNA in 3-day-old Drosophila larvae reared with control medium or medium supplemented with total pollen RNA (c) or the synthetic miRNA pool (d). Error bars represent SEM. ***p < 0.001, Student’s t test. (TIF) [file pgen.1006946.s009.tif]

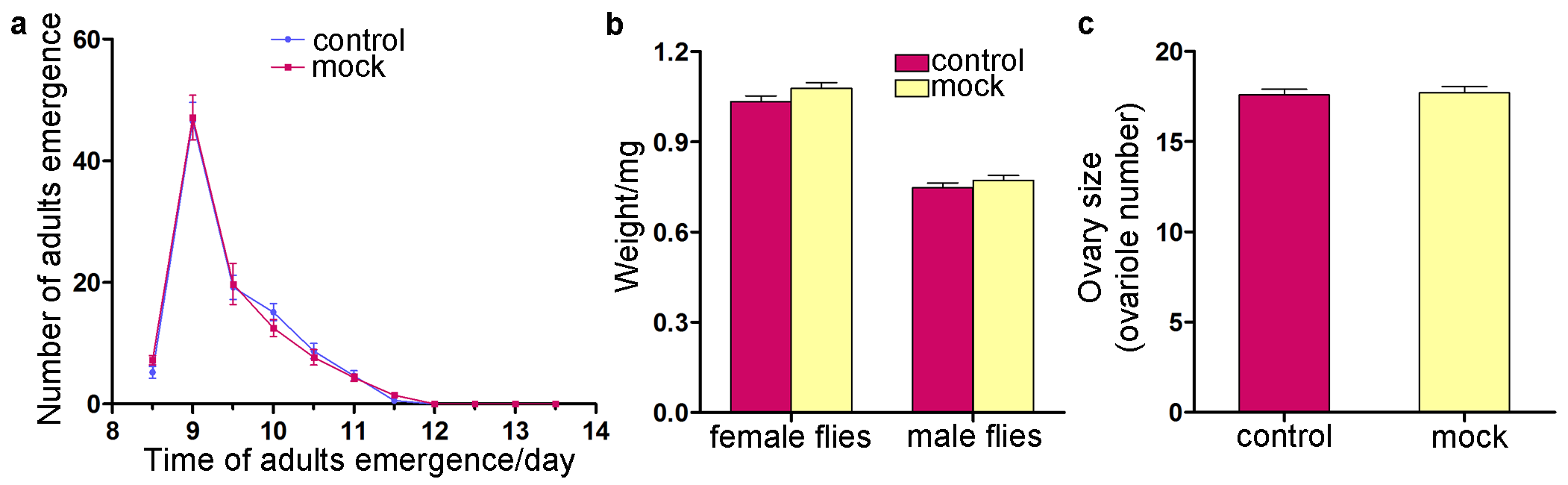

Supplement: S10 Fig — H2O instead of pollen was processed for RNA isolation and added to the diet as a mock control. Developmental time (a), body weight (b) and ovary size (c) of Drosophila that were reared with control medium or mock medium (n = 25–30). Error bars represent SEM. Student’s t test. (TIF) [file pgen.1006946.s010.tif]

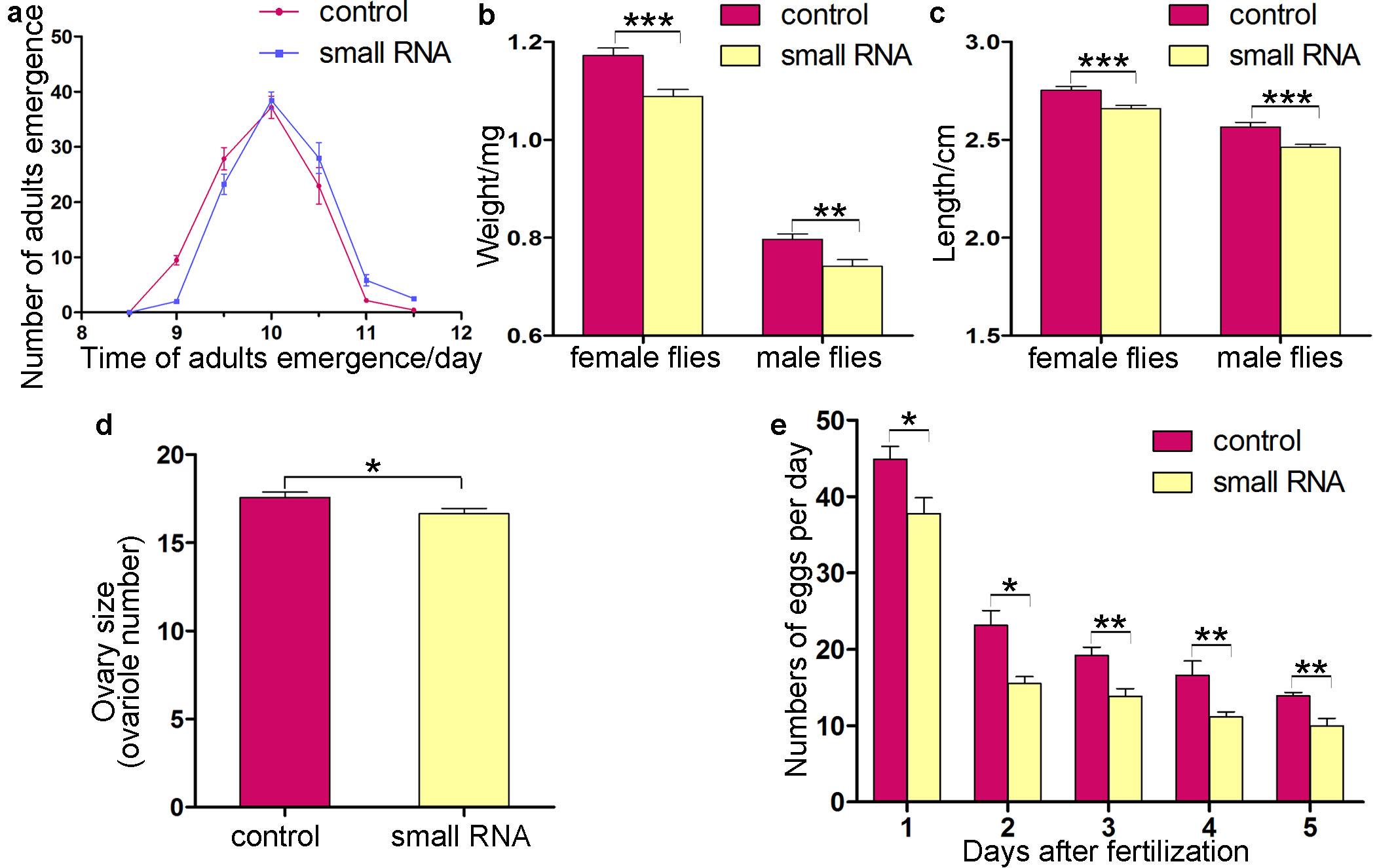

Supplement: S11 Fig — Developmental time (a), body weight (b), body length (c), ovary size (d) and fecundity (e) of Drosophila reared with control medium or medium containing small pollen RNA (n = 25–35). Error bars represent SEM. *p < 0.01; **p < 0.05; ***p < 0.001, Student’s t test. (TIF) [file pgen.1006946.s011.tif]

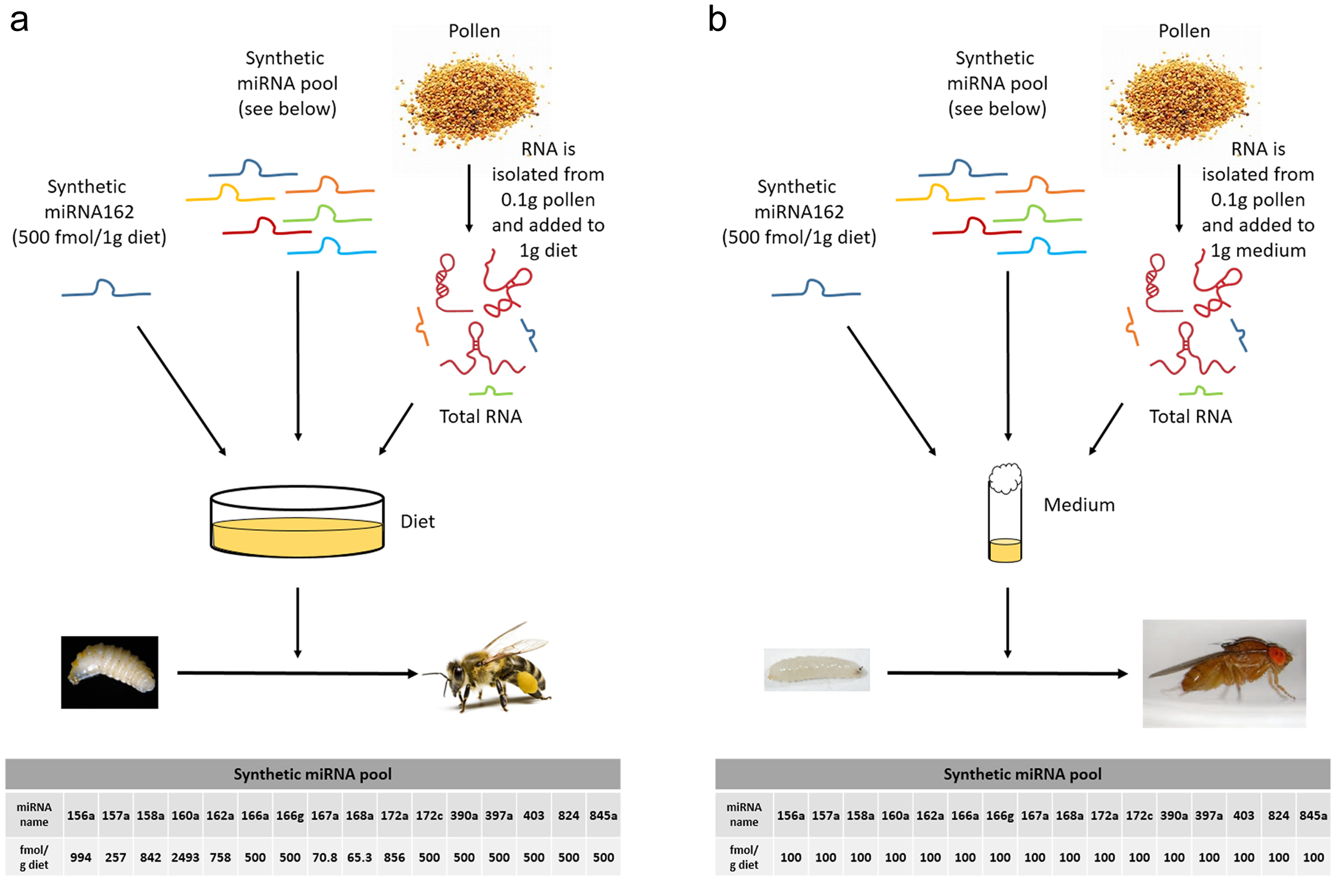

Supplement: S12 Fig — The detailed experimental procedure for preparation of the diets for honeybee (a) and Drosophila (b). (TIF) [file pgen.1006946.s012.tif]
