# Supplementary material for: Plant microRNAs in larval food regulate honeybee caste development
Source: PLoS Genet. 2017 Aug 31;13(8):e1006946. doi: 10.1371/journal.pgen.1006946 (PMC5578494; doi:10.1371/journal.pgen.1006946)
Supplement: S1 Table — (PDF) [file pgen.1006946.s013.pdf]

Table S1. Data for Illumina deep-sequencing of royal jelly, honey, pollen and beebread collected during *Brassica campestris* flowering stage.

Sequence	Plant miRNAs	RJ	Honey	Beebread	Pollen
UGACAGAAGAGAGUGAGCAC	ath-miR156a	3990	32521	182464	673018
UCCCAAAUGUAGACAAAGCA	ath-miR158a	455	1547	115410	26982
UCGCUUGGUGCAGGUCGGGAA	ath-miR168a	126	4624	5046	23374
UGAAGCUGCCAGCAUGAUCUA	ath-miR167a	104	4137	6267	6602
AGAAUCUUGAUGAUGCUGCAU	ath-miR172a	60	1129	8296	5266
UCGGACCAGGCUUCAUCCCCC	ath-miR165a	652	1231	10323	1872
UCGGACCAGGCUUCAUUCCCC	ath-miR166a	926	1928	5758	1860
UUGACAGAAGAUAGAGAGCAC	ath-miR157a	613	585	726	1735
UGACAGAAGAGAGAGAGCACA	osa-miR156k	14	32	430	1711
AGAAUCUUGAUGAUGCUGCAG	ath-miR172c	17	483	1875	435
UUAGAUUCACGCACAAACUCG	ath-miR403	1	15	184	413
UGCCUGGCUCCCUGUAUGCCA	ath-miR160a	48	3884	7534	332
CGGCUCUGAUACCAAUUGAUG	ath-miR845a	5	60	775	233
UGACAGAAGAUAGAGAGCAC	ath-miR157d	17	34	5	214
UAGACCAUUUGUGAGAAGGGA	ath-miR824	4	121	782	205
CGACAGAAGAGAGUGAGCAC	ath-miR156g	9	28	53	138
UCGGACCAGGCUUCAUUCCUC	osa-miR166g	213	165	218	134
UCGAUAAACCUCUGCAUCCAG	ath-miR162a	7	48	362	123
GCAGCACCAUUAAGAUUCAC	ath-miR172b*	0	4	4	67
UAUGAGAGUAUUAUAAGUCAC	ath-miR400	0	23	704	52
UGAAGCUGCCAGCAUGAUCUG	osa-miR167d	3	71	55	46
UGACAGAAGAAAGAGAGCAC	ath-miR156h	10	89	40	41
AAGCUCAGGAGGGAUAGCGCC	ath-miR390a	7	176	6	41
UGAAGCUGCCAGCAUGAUCUGG	ath-miR167d	2	35	55	40
UUCCACAGCUUUCUUGAACUG	ath-miR396a	1	9	27	30
UGACAACGAGAGAGAGCACGC	osa-miR535	6	15	1	28
UCGAACCAGGCUUCAUUCCCC	osa-miR166e	14	0	86	25
CAGCCAAGGAUGACUUGCCGA	ath-miR169a	1	3	27	24
UCAUUGAGUGCAGCGUUGAUG	ath-miR397a	2	92	169	16
UCGGACCAGGCUUCAUUCCCU	osa-miR166m	11	23	52	12
UUGGCAUUCUGUCCACCUCC	ath-miR394a	0	4	16	12
CCCCAAAUGUAGACAAAGCA	ath-miR158b	1	3	63	11
UGCCUGGCUCCCUGUAUGCCG	osa-miR160e	2	66	77	10
UUUCGUUGUCUGUUCGACCUU	ath-miR858	0	1	0	9
CAGCCAAGGAUGACUUGCCGG	ath-miR169b	0	6	3	7
UCGCUUGGUGCAGAUCGGGAC	osa-miR168a	38	162	1	6

GGAAUCUUGAUGAUGCUGCAU	ath-miR172e	1	9	38	5
UGCCAAAGGAGAUUUGCCCGG	ath-miR399f	3	80	36	5
UAGCCAAGAAUGACUUGCCUA	osa-miR169o	0	0	0	5
UUAGAUGACCAUCAACAAACU	ath-miR827	0	1	91	3
UGGAAGGGGCAUGCAGAGGAG	osa-miR528	0	723	0	2
UGGAGAAGCAGGGCACGUGCG	ath-miR164c	0	1	0	2
UUGGACUGAAGGGAGCUCCUU	ath-miR319c	0	0	0	2
UUUUGUAUGUUGAAGGUGUAU	ath-miR857	0	2	7	1
UUGGACUGAAGGGAGCUCCCU	ath-miR319a	0	1	5	1
UUCCACAGCUUUCUUGAACUU	ath-miR396b	0	0	3	1
UGGAGAAGCAGGGCACGUGCA	ath-miR164a	1	11	0	1
GUCCUCGGGAUGCGGAUUACC	ath-miR2111a*	0	4	0	1
UAGCCAAGGAUGACUUGCCUG	ath-miR169h	0	0	0	1
UGAGCCAAGGAUGACUUGCCG	ath-miR169d	0	3	1	1
UAGCCAAGGAUGACUUGCCGG	osa-miR169e	0	2	0	1
AUGCACUGCCUCUUCCCUGGC	ath-miR408	1	65	20	1
CUGCACUGCCUCUUCCCUGGC	osa-miR408	0	2	1	1
UGCCAAAGGAGAUUUGCCCCG	ath-miR399d	1	2	7	0
UCAAUGCAUUGAAAGUGACUA	ath-miR161.2	0	0	5	0
UGUGUUCUCAGGUCACCCCUG	ath-miR398b	0	5	2	0
UGAUUGAGCCGCGCCAAUAUC	ath-miR171a	0	1	2	0
UUAUUGAGUGCAGCGUUGAUG	osa-miR397b	0	0	2	0
CGACAGAAGAGAGUGAGCAUA	osa-miR156l	1	0	2	0
UCGGAUCAGGCUUCAUUCCUC	osa-miR166i	1	1	1	0
UCAUUGAGUGCAUCGUUGAUG	ath-miR397b	0	0	1	0
UAGGAUUCAAUCCUUGCUGCU	osa-miR1425	0	0	1	0
UCCAAAGGGAUCGCAUUGAUCC	ath-miR393a	0	1	1	0
UCCAAAGGGAUCGCAUUGAUC	osa-miR393	0	1	1	0
UGAAUCUUGAUGAUGCUGCAC	osa-miR172c	0	0	1	0
UUGAGCCGUGCCAAUAUCACG	ath-miR171c	0	0	1	0
UGAUUGAGCCGUGCCAAUAUC	osa-miR171b	0	0	1	0
UGCCAAAGGAGAUUUGCCCAG	osa-miR399f	0	3	0	0
UGCCAAAGGAGAUUUGCCUCG	ath-miR399e	0	0	0	0
UCGGACCAGGCUUCAAUCCCU	osa-miR166k	0	10	0	0
UAAUCUGCAUCCUGAGGUUUA	ath-miR2111a	0	7	0	0
UUUGGAUUGAAGGGAGCUCUG	osa-miR159a.1	1	6	0	0
UUUGGAUUGAAGGGAGCUCUU	ath-miR159b	0	4	0	0
UUUGGAUUGAAGGGAGCUCUA	ath-miR159a	0	4	0	0
UUUGGAUUGAAGGGAGCUCCU	ath-miR159c	0	4	0	0
UCGGCCUCGUGGAUGGACCAG	osa-miR820a	0	2	0	0
UGCCAAAGGAGAGUUGCCCUG	ath-miR399b	0	1	0	0
CGGUCUUGAGGCAGGAACUGAG	osa-miR1861h	0	1	0	0
UCAACAUGGUAUCAGAGCUGGAAG	osa-miR1873	0	1	0	0

GUGUUUGGUUUAGGGAUGAGGUGG	osa-miR1879	0	1	0	0
AGCUCUGAUACCAUGUUAGAUUAG	osa-miR1863	0	1	0	0
UCCAAAGGGAUCGCAUUGAUCU	osa-miR393b	0	1	0	0
UGCAGUUGUUGUCUCAAGCUU	osa-miR444b.2	0	1	0	0
UGGAGAAGCAGGGCACGUGAG	osa-miR164e	0	1	0	0
AGCGCCCAAGCGGUAGUUGUC	osa-miR1423	0	1	0	0
AUUGGAUUGAAGGGAGCUCCA	osa-miR159c	0	0	0	0
AUUGGAUUGAAGGGAGCUCCG	osa-miR159d	0	0	0	0
AUUGGAUUGAAGGGAGCUCCU	osa-miR159e	0	0	0	0
UGUGUUCUCAGGUCACCCCUU	ath-miR398a	0	0	0	0
AGGCAACUACACGUUGGGCGCUCG	osa-miR1423-5p.2	0	0	0	0
CAACUACACGUUGGGCGCUCGA	osa-miR1423b	0	0	0	0
CUGAAGUGUUUGGGGGGACUC	ath-miR395b	1	0	0	0
UUCGCUUGCAGAGAGAAAUCAC	ath-miR173	1	0	0	0
Plant miRNA reads		7371	54318	348124	745158
total RNA reads		10^7	10^7	10^7	10^7

Sequence	Animal miRNAs	RJ	Honey	Beebread	Pollen
UGAGGUAGUAGGUUGUAUAGUU	cel-let-7	601	18	573	523
UGGAAUGUAAAGAAGUAUGUA	cel-miR-1	72	2	66	93
UGAGGUAGUAGGUUGUAUAGU	ame-let-7	68	15	58	63
UGGAAUGUAAAGAAGUAUGGAG	ame-miR-1	90	442	18	8
UCAGGUACCUGAAGUAGCGCGCG	ame-miR-275	852	84	1	5
UGACUAGAUACAUACUCGUCU	ame-miR-996	9	2	0	4
UUGGUCCCCUUCAACCAGCUGU	ame-miR-133	7	0	6	3
UAGGAACUUCAUACCGUGCUCU	ame-miR-276	41	40	5	3
UGGACGGAGAACUGAUAAGGGC	ame-miR-184	228	74	0	2
ACCCUGUAGAUCCGAAUUUGU	ame-miR-10	5	0	2	2
ACCCUGUAGAUCCGAAUUUGUU	dme-miR-10-5p	5	0	2	2
UCCCUGAGACCCUAACUUGUGA	dme-miR-125	1	1	2	1
UAAUACUGUCAGGUAAAGAUGUC	ame-miR-8	35	20	0	1
UAAGGCACGCGGUGAAUGCCAAG	ame-miR-124	3	5	0	1
UGGCAGUGUGGUUAGCUGGUUGUG	dme-miR-34	0	0	0	1
GGCAAGAUGUCGGCAUAGCUGA	ame-miR-31a	53	30	1	0
UAUCACAGCCAUUUUUGACGAUU	ame-miR-13b	0	4	1	0
GUGAGCAAAGUUUCAGGUGU	ame-miR-87	7	8	1	0
GUGAGCAAAGUUUCAGGUGUGC	cel-miR-87	0	0	1	0
UGAACACAGCUGGUGGUAUCUCAGU	ame-miR-317	28	28	0	0
AUAAGUACUAGUGCCGCAGGAG	ame-miR-252	7	10	0	0
UUGUGCGUGUGACAGCGGCUA	ame-miR-210	0	8	0	0

AUUGCACUUGUCCCGGCCUAU	ame-miR-92a	12	7	0	0
UUUGUUCGUUCGGCUCGAGUUA	ame-miR-375	25	6	0	0
GUAAAUGGCACUGGAAGAAUUCAC	ame-miR-263	4	5	0	0
GUUAAUGGCACUGGAAGAAUUCAC	dme-miR-263a	4	5	0	0
UAUCACAGCCAGCUUUGAUGAGC	ame-miR-2	14	5	0	0
CUAAGUACUAGUGCCGCAGGAG	dme-miR-252	1	3	0	0
UCAGUCUUUUUCUCUCUCCUA	ame-miR-14	14	2	0	0
UGACUAGAUCCACACUCAUUAA	ame-miR-279	6	2	0	0
CAAAUUCGGUUCUAGAGAGGUUU	dme-miR-10-3p	10	1	0	0
UGAAAGACAUGGGUAGUGA	ame-miR-71	7	1	0	0
UGGCAGUGUUGUUAGCUGGUUG	ame-miR-34	3	1	0	0
CAGCGAGGUAUAGAGUUCCUACG	dme-miR-276*	2	1	0	0
UGAGAUCAUUGUGAAAGCUGAUU	ame-bantam	1	1	0	0
UAAAGCUAGAUUACCAAAGCA	ame-miR-79	1	1	0	0
GAAGCUCGUCUCUACAGGUAUCU	ame-miR-993	18	1	0	0
AAUUGCACCCGUCCCGGCCUGA	ame-miR-92b	4	1	0	0
CCCCUGAGACCCUAACUUGUGA	ame-miR-125	1	1	0	0
UGAGUAUUACAUCAGGUACUGGU	ame-miR-12	1	1	0	0
UAAGGCACGCGGUGAAUGCCA	cel-miR-124	1	1	0	0
UGGCAAGAUGUCGGCAUAGCUGA	dme-miR-31a	1	1	0	0
UGAGAUCGUUCAGUACGGCAAU	cel-miR-58	0	1	0	0
UGAGGUAGGCUCAGUAGAUGCGA	cel-miR-48	0	1	0	0
UAAAGCUAGAUUACCAAAGCAU	dme-miR-79	1	0	0	0
AAAUAUCAGCUGGUAAUUCU	ame-miR-283	0	0	0	0
CAUUGCACUUGUCCCGGCCUAU	dme-miR-92a	0	0	0	0
UGAACACAGCUGGUGGUAUCCAGU	dme-miR-317	0	0	0	0
UAAAUAUCAGCUGGUAAUUCU	dme-miR-283	0	0	0	0
UAUCACAGCCAGCUUUGAUGUGC	cel-miR-2	0	0	0	0
UAUCACAGCCAGCUUUGAUGGGC	dme-miR-2c	0	0	0	0
AACCCGUAGAUCCGAACUUGUG	ame-miR-100	3	0	0	0
UCUUUGGUUAUCUAGCUGUAUGA	ame-miR-9a	2	0	0	0
UAAAUGCACUAUCUGGUACGACA	ame-miR-277	1	0	0	0
AUUGUACUUCAUCAGGUGCUCUG	ame-miR-305	1	0	0	0
UAUCACAGCCAUUUUGAUGAG	ame-miR-13a	1	0	0	0
AGAUAUGUUUGAUAUUCUUGGUUGUU	ame-miR-190	1	0	0	0
AGAUAUGUUUGAUAUUCUUGGUUG	dme-miR-190	1	0	0	0
Animal miRNA reads		2253	840	737	712
total RNA reads		10^7	10^7	10^7	10^7
