# Supplementary material for: Plant microRNAs in larval food regulate honeybee caste development
Source: PLoS Genet. 2017 Aug 31;13(8):e1006946. doi: 10.1371/journal.pgen.1006946 (PMC5578494; doi:10.1371/journal.pgen.1006946)
Supplement: S2 Table — (PDF) [file pgen.1006946.s014.pdf]

Table S2. Data for Illumina deep-sequencing of royal jelly, honey, pollen and beebread collected during *Camellia japonica* flowering stage.

Sequence	Plant miRNAs	RJ	Honey	Beebread	Pollen
UCGGACCAGGCUUCAUUCCCC	ath-miR166a-3p	155	1586	25825	50041
UCGGACCAGGCUUCAUUCCCU	osa-miR166m	15	321	4064	9728
UCGAUAAACCUCUGCAUCCAG	ath-miR162a-3p	2	2607	3516	4005
UCGGACCAGGCUUCAUUCCUC	osa-miR166h-3p	17	247	922	1986
UGACAGAAGAGAGUGAGCAC	ath-miR156a-5p	7	1187	808	907
GUUCAAUAAAGCUGUGGGAAG	ath-miR396a-3p	0	46	269	869
UUAGAUUCACGCACAAACUCG	ath-miR403-3p	2	658	352	841
UUCCACAGCUUUCUUGAACUU	ath-miR396b-5p	1	212	263	702
UUCCACAGCUUUCUUGAACUG	ath-miR396a-5p	1	147	160	679
UUGACAGAAGAUAGAGAGCAC	ath-miR157a-5p	0	75	85	585
UGAAGCUGCCAGCAUGAUCUG	osa-miR167d-5p	0	61	627	492
UCGCUUGGUGCAGGUCGGGAA	ath-miR168a-5p	0	93	299	391
UCCACAGGCUUUCUUGAACUG	osa-miR396e-5p	6	1	781	384
UCUCCACAGGCUUUCUUGAACU	osa-miR396f-5p	6	0	781	383
UCGGACCAGGCUUCAUCCCCC	ath-miR165a-3p	2	754	112	329
UGAAGCUGCCAGCAUGAUCUGG	ath-miR167d	0	49	197	245
GUUCAAUAAAGCUGUGGGAA	osa-miR396a-3p	0	11	67	238
CCCGCCUUGCAUCAACUGAAU	ath-miR168a-3p	0	36	85	157
UGACAGAAGAUAGAGAGCAC	ath-miR157d	0	7	3	110
UUGGACUGAAGGGUGCUCCC	osa-miR319b	2	0	226	107
GGAAUGUUGUCUGGCUCGAGG	osa-miR166d-5p	0	86	211	93
UGCCUGGCUCCCUGUAUGCCA	ath-miR160a-5p	0	114	14	84
UGAAGCUGCCAGCAUGAUCUA	ath-miR167a-5p	0	78	42	73
UUGGACUGAAGGGAGCUCCCU	ath-miR319a	6	43	20	67
UCCACAGGCUUUCUUGAACGG	osa-miR396d	2	0	90	61
GCGUAUGAGGAGCCAUGCAUA	ath-miR160a-3p	0	2	16	34
UCGAACCAGGCUUCAUUCCCC	osa-miR166e-3p	0	1	12	34
GGAAUCUUGAUGAUGCUGCAU	ath-miR172e-3p	0	2	16	29
UGCCUGGCUCCCUGUAUGCCG	osa-miR160e-5p	0	47	7	29
UGACAGAAGAGAGAGAGCAC	ath-miR156j	0	14	2	21
UUGGACUGAAGGGAGCUCCUU	ath-miR319c	28	34	4	21
UGACAACGAGAGAGAGCACGC	osa-miR535-5p	0	2	3	19
AGAAUCUUGAUGAUGCUGCAU	ath-miR172a	0	5	12	16
GGAAUGUUGUCUGGCUCGGGG	osa-miR166b-5p	0	6	3	14
UCGCUUGGUGCAGAUCGGGAC	osa-miR168a-5p	1	0	15	12
UCGAUAAGCCUCUGCAUCCAG	osa-miR162b	0	7	9	11

UGUGUUCUCAGGUCACCCCUU	ath-miR398a-3p	0	0	5	10
CUGAAGUGUUUGGGGGAACUC	ath-miR395a	0	19	5	10
AGAAUCUUGAUGAUGCUGCAG	ath-miR172c	0	34	0	9
GCUCACUUCUCUUUCUGUCAGC	osa-miR156f-3p	0	7	6	8
CUGCACUGCCUCUUCCCUGGC	osa-miR408-3p	0	2	41	8
UUUGGAUUGAAGGGAGCUCUA	ath-miR159a	3	43	2	7
GAUCCCCGGCAACGGCGCCA	ath-miR8175	20	129	0	7
CGGCUCUGAUACCAAUUGAUG	ath-miR845a	11	927	5	7
UGUGUUCUCAGGUCGCCCCUG	osa-miR398b	0	4	5	7
GCUCACUGCUCUAUCUGUCAGA	ath-miR156c-3p	0	11	3	7
UUUGGAUUGAAGGGAGCUCCU	ath-miR159c	0	7	0	6
AUGCACUGCCUCUUCCCUGGC	ath-miR408-3p	0	11	2	5
UGCCUGGCUCCCUGAAUGCCA	osa-miR160f-5p	0	0	3	5
UUUGGAUUGAAGGGAGCUCUG	osa-miR159b	1	12	3	5
UCGGAUCAGGCUUCAUUCCUC	osa-miR166i-3p	0	0	1	4
UGCAGUUGUUGCCUCAAGCUU	osa-miR444f	0	0	4	4
UUUGGAUUGAAGGGAGCUCUU	ath-miR159b-3p	1	13	1	4
GCUCACUGCUCUUUCUGUCAGA	ath-miR156a-3p	0	0	16	4
UGCAGUUGUUGUCUCAAGCUU	osa-miR444b.2	0	0	3	4
ACUGAACUCAAUCACUUGCUGC	osa-miR5538	0	1	1	4
UGGAAGGGGCAUGCAGAGGAG	osa-miR528-5p	0	0	9	3
GGAAUGUUGGCUGGCUCGAGG	osa-miR166h-5p	0	1	3	3
CGACAGAAGAGAGUGAGCAUA	osa-miR156l-5p	0	1	3	3
UGAGGAGGAACAUAUUUACUAG	osa-miR5523	0	0	1	2
AUCAUGCGAUCUCUUUGGAUU	ath-miR393b-3p	0	0	26	2
UGGAGGCAGCGGUUCAUCGAUC	ath-miR162a-5p	0	2	2	2
UGAUUGAGCCGCGCCAAUAUC	osa-miR171a	0	0	0	1
GCUCACUUCUCUCUCUGUCAGC	osa-miR156c-3p	0	0	4	1
AGACUACAAUUAUCUGAUCA	osa-miR5083	0	1	0	1
AUGGAAUAUAUGACAAAGGUGG	osa-miR5532	0	0	0	1
UCCAAAGGGAUCGCAUUGAUCU	osa-miR393b-5p	0	0	1	1
UCAUUGAGUGCAGCGUUGAUG	ath-miR397a	0	0	3	1
UGACAGAAGAAAGAGAGCAC	ath-miR156h	0	4	0	1
UCCCAAAUGUAGACAAAGCA	ath-miR158a-3p	4	6393	3	1
UUGAGCCGUGCCAAUAUCACG	ath-miR171b-3p	0	2	0	1
UUGGCAUUCUGUCCACCUCC	ath-miR394a	0	34	2	1
AUGAUGAUGAUGAUGAUGAAA	ath-miR5658	7	3	0	1
UGCAGUUGCUGCCUCAAGCUU	osa-miR444a-3p.2	0	0	1	1
GCAGCACCAUCAAGAUUCAC	osa-miR172d-5p	0	0	0	1
UGACAGAAGAGAGAGAGCACA	osa-miR156k	0	0	1	1
UUUUGGAACGGAGUGAGUAUU	osa-miR1439	0	0	0	1
UCGGCCUCGUGGAUGGACCAG	osa-miR820a	0	0	0	1
AGGUCAUGCUGUAGUUUCAUC	osa-miR167h-3p	0	0	5	1

UCCAAAGGGAUCGCAUUGAUCC	ath-miR393a-5p	0	0	1	0
UCCAAAGGGAUCGCAUUGAUC	osa-miR393a	0	0	1	0
CGACAGAAGAGAGUGAGCAC	ath-miR156g	0	5	6	0
UUAUUGAGUGCAGCGUUGAUG	osa-miR397b	0	0	2	0
AUUGGAUUGAAGGGAGCUCCG	osa-miR159d	0	1	0	0
AUUGGAUUGAAGGGAGCUCCU	osa-miR159e	0	1	0	0
AUUGGAUUGAAGGGAGCUCCA	osa-miR159c	0	1	0	0
UAUUGGCCUGGUUCACUCAGA	ath-miR171a-5p	0	2	0	0
UGCCAAAGGAGAGUUGCCCUG	ath-miR399b	0	3	1	0
UAAUCUGCAUCCUGAGGUUUA	ath-miR2111a-5p	0	1	0	0
UUUCGUUGUCUGUUCGACCUU	ath-miR858a	0	11	2	0
GCUCUCUAGCCUUCUGUCAUC	ath-miR157a-3p	0	3	0	0
GCUCUCUAUACUUCUGUCACC	ath-miR157c-3p	0	2	2	0
GGAAUGUUGUCUGGAUCGAGG	ath-miR165a-5p	0	2	0	0
UGUGUUCUCAGGUCACCCCUG	ath-miR398b-3p	0	3	0	0
GCAAGUUGACCUUGGCUCUGC	ath-miR169f-3p	0	0	0	0
CUUUGUCUACAAUUUUGGAAA	ath-miR158a-5p	0	6	0	0
GGACUGUUGUCUGGCUCGAGG	ath-miR166a-5p	0	1	0	0
UCCGGCAAGUUGACCUUGGCU	ath-miR169g-3p	0	0	0	0
UGCCAAAGGAGAUUUGCCCUG	ath-miR399a	0	1	0	0
AUGGUCGAAGUAGGCAAAAUC	ath-miR472-5p	0	2	0	0
AAGCUCAGGAGGGAUAGCGCC	ath-miR390a-5p	0	1	0	0
CGCUAUCCAUCCUGAGUUCC	ath-miR390b-3p	0	2	0	0
CUGAAGUGUUUGGGGGGACUC	ath-miR395b	0	9	0	0
UGACAGAAGAGAGAGAGCAG	ath-miR156i	0	0	0	0
GGAAUGUUGUCUGGCACGAGG	ath-miR166e-5p	0	9	0	0
UAGACCAUUUGUGAGAAGGGA	ath-miR824-5p	0	4	0	0
CCUUCUCAUCGAUGGUCUAGA	ath-miR824-3p	0	5	0	0
UGAAAGUGACUACAUCGGGGU	ath-miR161.1	0	51	0	0
CCCCAAAUGUAGACAAAGCA	ath-miR158b	0	24	0	0
CGCUAUCCAUCCUGAGUUUCA	ath-miR390a-3p	0	1	1	0
GCUCACUCUCUAUCCGUCACC	ath-miR156f-3p	0	1	0	0
UUCGUUGUCUGUUCGACCUUG	ath-miR858b	0	4	2	0
UUAGAUGACCAUCAACAAACU	ath-miR827	0	1	0	0
ACAGGGAACAAGCAGAGCAUG	ath-miR408-5p	0	4	0	0
UGCUCACCUCUCUUUCUGUCAGU	ath-miR156b-3p	0	12	0	0
GCUCAAGAAAGCUGUGGGAAA	ath-miR396b-3p	0	3	0	0
GUGAAGUGUUUGGGGGAACUC	osa-miR395d	0	2	0	0
UUGCUGCCUCAAGCUUGCUGC	osa-miR444a-3p.1	0	1	0	0
UGAUUGAGCCGUGCCAAUAUC	osa-miR171b	0	2	0	0
UGCCAAAGGAGAAUUGCCCUG	osa-miR399a	0	1	0	0
GUUCGCGUCGGGUUCACCA	osa-miR5077	0	15	0	0
CUUGGAUUGAAGGGAGCUCUA	osa-miR159f	0	6	0	0

GUGAAGUGUUUGGAGGAACUC	osa-miR395c	0	1	0	0
UGCCAAAGGAGAGUUGCCCUA	osa-miR399j	0	1	0	0
AUGAAGUGUUUGGAGGAACUC	osa-miR395o	0	1	0	0
UCGGACCAGGCUUCAAUCCCU	osa-miR166k-3p	0	0	3	0
GAUCCCGCCUUGCACCAAGUGAAU	osa-miR168a-3p	0	0	1	0
AGCUCUGAUACCAUGUUAGAUUAG	osa-miR1863a	0	0	1	0
GGUCAAGAAAGCUGUGGGAAG	osa-miR396c-3p	0	0	1	0
CAGGGAUGAGGCAGAGCAUGG	osa-miR408-5p	0	0	3	0
UGGAGAAGCAGGGUACGUGCA	osa-miR164c	0	0	1	0
CGAUUCCCCAGCGGAGUCGCCA	osa-miR5072	3	0	0	0
Plant miRNA reads		303	16406	40116	73954
total RNA reads		10^7	10^7	10^7	10^7

Sequence	Animal miRNAs	RJ	H	BB	P
UGGACGGAGAACUGAUAAGGGC	ame-miR-184	21480	1561	70	97
AUACGGUGAUGAGUUUAAACAG	ame-miR-3720	18775	254	1	7
UCUUUGGUUAUCUAGCUGUAUGA	ame-miR-9a	9632	1492	106	163
UAGGAACUUCAUACCGUGCUCU	ame-miR-276	6116	10100	368	452
GGCAAGAUGUCGGCAUAGCUGA	ame-miR-31a	5640	341	11	6
UGGAAUGUAAAGAAGUAUGGAG	ame-miR-1	5051	2513	130	392
UGAGAUCAUUGUGAAAGCUGAUU	ame-bantam	4907	459	100	166
UAAUACUGUCAGGUAAAGAUGUC	ame-miR-8	4675	1151	18	43
CCAGAUCUAACUCUUCCAGCUC	ame-miR-750	1565	476	30	31
AACCCGUAGAUCCGAACUUGUG	ame-miR-100	1471	741	65	81
UUUUGAUUGUUGCUCAGAAAGC	ame-miR-315	1467	53	18	22
UCAGGUACUGAGUGACUCUGAG	ame-miR-306	1315	71	0	1
ACCCUGUAGAUCCGAAUUUGU	ame-miR-10	966	162	38	59
ACCCUGUAGAUCCGAAUUUGUU	dme-miR-10-5p	966	162	38	59
CAUCUUACCGGGCAGCAUUAGA	dme-miR-8-5p	939	246	9	3
UCGGUGGGACUUUCGUCCGUUU	ame-miR-278	800	82	0	3
AUGCAAAAGCUAGAACUCAUAGA	ame-miR-6052	714	16	0	2
GUAAAUGGCACUGGAAGAAUUCAC	ame-miR-263a	658	218	34	44
AAUGGCACUGGAAGAAUUCACGGG	dme-miR-263a-5p	658	218	34	44
AUUGUACUUCAUCAGGUGCUCUG	ame-miR-305	477	313	1	9
UACCCUGUAACGUCCUGAGACU	ame-miR-3785	457	33	0	0
AAAUAUCAGCUGGUAAUUCU	ame-miR-283	342	267	3	13
AAAUAUCAGCUGGUAAUUCUGG	dme-miR-283-5p	342	267	3	13
UCAGGUACCUGAAGUAGCGCGCG	ame-miR-275	341	540	1	10
UAAGCUCUGUGUACUUUUUAC	ame-miR-6037	297	35	0	0

UUUGUUCGUUCGGCUCGAGUUA	ame-miR-375	282	178	1	5
AUAUGAACUCUUAUGUACGUGA	ame-miR-6005-5p	192	8	0	0
CUUGUCCUGGUUCAUGUAGGGCG	ame-miR-3786	183	9	0	1
UUAUGAUCUGGAAUACUAGG	ame-miR-9893	158	0	0	0
GAAGCUCGUCUCUACAGGUAUCU	ame-miR-993	136	15	0	1
CAUCACAGGCAGAGUUCUAGUU	ame-miR-11	121	186	0	2
UGUCAUGGAGUUGCUCUCUUUGU	ame-miR-281	117	182	1	3
UGAAAGACAUGGGUAGUGA	ame-miR-71	116	43	2	4
UUUGUGACUGUAACAACAAAUG	ame-miR-6057	116	0	0	0
UGAGGUAGUAGGUUGUAUAGU	ame-let-7	115	192	52	64
CUUGGCACUGGAAGAAUUCAC	ame-miR-263b	111	59	40	39
CGUGAUGUGACGUAGUGGUUCU	ame-miR-989	110	40	0	0
UGACUAGAUCCACACUCAUCCA	ame-miR-279d	78	82	18	26
UGACUAGAUACAUACUCGUCU	ame-miR-996	72	10	0	0
UGACUAGAUCGAAAUACUCGUCCC	ame-miR-279b	66	11	0	1
AAUUGCACCCGUCCCGGCCUGA	ame-miR-92b	62	10	0	0
GACUCACGUCGACUGGGUGUCCGC	ame-miR-3759	61	25	0	1
UAAUCUCAUGCGGUAACUGUGAG	ame-miR-3477	60	170	0	7
AUAAGUACUAGUGCCGCAGGAG	ame-miR-252a	60	42	0	0
ACGUAUACUGAAUGUAUCCUGA	ame-miR-iab-4	57	2	0	0
UCAGUCUUUUUCUCUCUCCUAU	dme-miR-14-3p	56	70	11	12
UAUCACAGCAGUAGUUACCUGGU	ame-miR-2944	56	30	1	1
UCAGUCUUUUUCUCUCUCCUA	ame-miR-14	55	68	12	12
AAAAUUGCGCACGCUCUGAAUAU	ame-miR-6051	54	11	0	1
UGGAAGACUAGUGAUUUUGUUGU	ame-miR-7	51	245	32	84
CAAAUUCGGUUCUAGAGAGGUUU	dme-miR-10-3p	49	86	3	4
GUGAGCAAAGUUUCAGGUGU	ame-miR-87	43	25	1	4
UAAAUGCACUAUCUGGUACGACA	ame-miR-277	42	38	3	1
UAGUACGGGCAGUACUGGGA	ame-miR-6040	42	6	0	0
UGACUAGAUCCACACUCAUUAA	ame-miR-279a	32	78	3	1
UUUCGAGGCUGUUUAAAUGGAUU	ame-miR-9864	31	2	0	1
UGAACACAGCUGGUGGUAUCUCAGU	ame-miR-317	31	23	2	3
UAUCACAGCCAUUUUUGACGAUU	ame-miR-13b	27	2	0	1
UGGCAAGAUGUCGGCAUAGCUGA	dme-miR-31a-5p	27	2	1	0
UAUCACAGCCAUUUUGAUGAG	ame-miR-13a	26	2	0	0
GUAGGCCGGCGGAAACUACUUGC	ame-miR-2796	21	17	4	5
CCCCUGAGACCCUAACUUGUGA	ame-miR-125	21	53	1	0
AGAUAUGUUUGAUAUUCUUGGUUGUU	ame-miR-190	20	35	6	4
AGAUAUGUUUGAUAUUCUUGGUUG	dme-miR-190-5p	20	37	6	4
UGAGUAUUACAUCAGGUACUGGU	ame-miR-12	18	7	0	0
UUUUAGAAUUCCUACGCUUUACC	ame-miR-927a	16	17	0	0
UUUAGAAUUCCUACGCUUUACC	dme-miR-927-5p	16	17	0	0
UAUCACAGCCAGCUUUGAUGAGC	ame-miR-2	15	16	2	6

CUGAUUUCUUUCAUAAGGAGGA	ame-miR-3756	13	0	0	0
UGACUAGAGUCACACUCGUCCA	ame-miR-279c	10	11	1	0
UCACCGGGUAGGAUUCAUCCA	ame-miR-3791	6	48	3	5
UCCCUGAGACCCUAACUUGUGA	dme-miR-125-5p	6	11	8	3
CUAAGUACUAGUGCCGCAGGAG	dme-miR-252-5p	6	8	2	1
UAAGCGUAUAGCUUUUUCCCUU	ame-miR-965-3p	6	0	0	0
UUCGGAAGAAUGUAGAGAAAAAG	ame-miR-9890	6	0	0	0
UUGGUCCCCUUCAACCAGCUGU	ame-miR-133	5	7	2	10
UUAAGUAGUAGUGUCGUAGAUGA	ame-miR-252b	4	24	0	0
GUAGGUAACGACUGAUGGGAAC	ame-miR-6001-5p	4	50	0	0
AAGAGGCUUCUUAGGCAAGUGAU	ame-miR-6044	4	2	0	0
GUGCAUUGUAGUUGCAUUG	ame-miR-33	4	0	0	0
UAAAGCUAGAUUACCAAAGCA	ame-miR-79	4	0	0	0
UAAAGCUAGAUUACCAAAGCAU	dme-miR-79-3p	4	0	0	0
UUAUUGCUUGAGAAUACACGUA	ame-miR-137	3	3	1	0
AUUGCACUUGUCCCGGCCUAU	ame-miR-92a	3	16	0	0
AUUCACUGGACGGCAAUGGGCU	ame-miR-6045	3	1	0	0
UCGUGACUUUCUUUUCAGCCCCU	ame-miR-3743	3	1	0	0
UUCUCUUUGGUUGUUACCACUA	ame-miR-6001-3p	3	16	0	0
UAUUGCUUGAGAAUACACGUAG	dme-miR-137-3p	3	3	1	0
UAGGAACGGUAGUAGUGGAGA	ame-miR-6042	3	0	0	0
UUCGUUGUCAACGAAACCUGCA	ame-miR-981	3	0	0	0
UCGGUAAGCAGAGUAUAAGACCU	ame-miR-3715	2	20	0	0
UAGGAACUUAAUACCGUGCUCU	dme-miR-276b-3p	2	3	0	0
UGAUUUAUGUGGUGAUUGUGC	ame-miR-9865	2	0	0	0
AUACGAAAGACCGCGCGGAUGU	ame-miR-6053	2	0	0	0
UGAACACAGCUGGUGGUAUCCAGU	dme-miR-317-3p	2	1	0	1
AUUGACUCUAGUAGGGAGUCC	ame-miR-929	2	0	0	0
AAAUUGACUCUAGUAGGGAGUC	dme-miR-929-5p	2	0	0	0
UGUUAACUGUAAGACUGUGUCU	dme-miR-999-3p	1	4	0	1
UACGGAUUGCGUGACUUUUCGA	ame-miR-3719	1	2	0	0
UAGCACCAUUUGAAAUCAGU	ame-miR-29b	1	7	0	0
UGGUAACUCCACCACCGUUGGC	ame-miR-2765-5p	1	19	0	0
AUGGUGACCGUGAUCUAUUCCA	ame-miR-6043	1	3	0	0
UCGGGAAGGUAGUUGCGGCGGAU	ame-miR-3049-5p	1	8	0	0
UCACAACCUUUUUGAGUGAGCGA	ame-miR-307	1	1	0	0
AAAUAUCAUCGAGAUCGAAGGA	ame-miR-6041	1	1	0	0
UUUUAGAAUUUGUACGCUCUGU	ame-miR-927b	1	1	0	0
UGUCUUUUUCCGCUUUGCUGCCG	ame-miR-316	1	7	0	0
UGGCAGUGUUGUUAGCUGGUUG	ame-miR-34	1	3	0	0
CAGCGAGGUAUAGAGUUCCUACG	dme-miR-276b-5p	1	2	0	0
UCGGCAAUGAUCGGACGUGGUC	ame-miR-9885	1	0	0	0
UCACUGGGUUAUGUUUGUCCC	ame-miR-318	1	0	0	0

UCGGUCGGUGACGAAGCUCCC	ame-miR-9884	1	0	0	0
UGGGAUGUUGAAUCUGUUGCAG	ame-miR-9878	1	0	0	0
UCGUGUCCGUUUCUCGUUUCGA	ame-miR-9895	1	0	0	0
UAAGGCACGCGGUGAAUGCCAAG	ame-miR-124	1	0	0	0
UAAGUGAUUGAUCGAUCGUGGAU	ame-miR-3781	1	0	0	0
UAGAGGGAGAGACGGAACCA	ame-miR-6059	1	0	0	0
GACGUUGCACGUUACCACGCCG	ame-miR-6061	1	0	0	0
UGACGCGAUUGUGGAAAUCG	ame-miR-9892	1	0	0	0
UGCGAGAUUUCUUUUCAGUCGUG	ame-miR-3747b	1	0	0	0
UAGGCGUCACGUUGUGGAACG	ame-miR-9886	1	0	0	0
AACCCGUAAAUCCGAACUUGUG	dme-miR-100-5p	1	0	0	0
UAUCACAGCCAGCUUUGAUGGGC	dme-miR-2c-3p	1	0	0	0
UGGCAGUGUGGUUAGCUGGUUGUG	dme-miR-34-5p	0	1	1	6
AAGAGAGCUAUCCGUCGACAGU	dme-miR-281-2-5p	0	0	3	4
UCAAUUCCGUAGUGCAUUGCAG	ame-miR-932	0	0	0	1
UCCCCUGUCCUGUCCCGAUAG	ame-miR-3718a	0	1	0	1
CUUGGCACUGGGAGAAUUCAC	dme-miR-263b-5p	0	0	0	1
CGUGGGUACUACUCGUCUCUAU	ame-miR-6000a-5p	0	3	0	0
AACGAGUGGUGGAUGCCAGCGU	ame-miR-3726	0	2	0	0
CAAUGCCCUUCGAAAUCCCAAA	ame-miR-2788	0	1	0	0
AAACGGAUCAAGCUUUUUGUGA	ame-miR-6067	0	1	0	0
UGAGAUUCACUCCUCCAACUUAC	ame-miR-1175	0	4	0	0
UCGACGAGAAUUCCGUGACCGGU	ame-miR-6046	0	1	0	0
UAGAGACGAGUAGUACCCACGAG	ame-miR-6000b	0	1	0	0
UGAGAUCAUUUUGAAAGCUGAUU	dme-bantam-3p	0	1	0	0
CAUCACAGUCUGAGUUCUUGC	dme-miR-11-3p	0	0	3	0
UCUUUGGUAUUCUAGCUGUAGA	dme-miR-9c-5p	0	0	1	0
Animal miRNA reads		93239	24221	1306	2052
total RNA reads		10^7	10^7	10^7	10^7
