# Supplementary material for: Plant microRNAs in larval food regulate honeybee caste development
Source: PLoS Genet. 2017 Aug 31;13(8):e1006946. doi: 10.1371/journal.pgen.1006946 (PMC5578494; doi:10.1371/journal.pgen.1006946)
Supplement: S3 Table — A list of orthologous genes of honeybee and Drosophila was examined for significant associations with specific GO functional categories based on the annotation of Drosophila genes. Among the 10 top-ranked GO categories, 6 are directly related to development process. As a control, none of the irrelevant GO process is related to development process. (PDF) [file pgen.1006946.s015.pdf]

Table S3. Functional analysis of gene lists using GO terms. A list of orthologous genes of honeybee and Drosophila was examined for significant associations with specific GO functional categories based on the annotation of Drosophila genes. Among the 10 top-ranked GO categories, 6 are directly related to development process. As a control, none of the irrelevant GO categories is related to development process.

GO functional categories	Total numbers of Drosophila genes in the GO terms (total number = 14217)	Numbers of examined genes in the GO terms (total number = 96)	P-value
The top 10 relevant GO process			
system development (GO:0048731)	2296	49	9.92E-12
biological regulation (GO:0065007)	3990	64	1.50E-11
single-multicellular organism process (GO:0044707)	3199	57	2.73E-11
multicellular organismal process (GO:0032501)	4088	64	5.24E-11
anatomical structure development (GO:0048856)	3197	56	1.32E-10
single-organism developmental process (GO:0044767)	3310	56	6.18E-10
multicellular organism development (GO:0007275)	2880	52	7.76E-10
developmental process (GO:0032502)	3351	56	1.06E-09
animal organ development (GO:0048513)	1321	34	8.13E-09
regulation of biological process (GO:0050789)	3615	56	2.86E-08
The top 10 irrelevant GO process			
biological_process (GO:0008150)	11306	88	1.00E+00
localization (GO:0051179)	2134	27	1.00E+00
metabolic process (GO:0008152)	4663	25	1.00E+00
transport (GO:0006810)	1695	23	1.00E+00
organelle organization (GO:0006996)	1729	23	1.00E+00
establishment of localization (GO:0051234)	1764	23	1.00E+00
nitrogen compound metabolic process	3624	23	1.00E+00

(GO:0006807)			
primary metabolic process (GO:0044238)	3849	23	1.00E+00
organic substance metabolic process (GO:0071704)	4182	23	1.00E+00
multi-organism process (GO:0051704)	1486	22	1.00E+00
