# Supplementary material for: Plant microRNAs in larval food regulate honeybee caste development
Source: PLoS Genet. 2017 Aug 31;13(8):e1006946. doi: 10.1371/journal.pgen.1006946 (PMC5578494; doi:10.1371/journal.pgen.1006946)
Supplement: S4 Table — (PDF) [file pgen.1006946.s016.pdf]

Table S4. Potential target genes for individual plant miRNAs.

Target gene of plant miRNAs: TOR

MIRNA	Position in target gene	Structure of binding site	minimum of free energy
ath-miR156a-5p	1687	U UGA UACA A--<br>GUGC UCAUUUUCU UCUG |||| |||:|:||| |||| CACG AGUGAGAGA AGAC<br>- --- ---- AGU	-22
ath-miR160a-5p	6109	C U C AGUUAUA U A G<br>UGGC GUAC GGGA G CCGGG CA |||| :||| ||||	||:|| || ACCG UAUG CCCU C GGUCC GU<br>- - U ------- - - -	-27.5
ath-miR162a-3p	3678	G AUG UA A U<br>CUGGA GC AGA GUUUGUCGA ||||| || ||| ||||:|||| GACCU CG UCU CAAAUAGCU<br>- A-- -- C -	-26.4
osa-miR166g-3p	5448	A UAU AUUG A<br>GAGG AUGAAGCU UCGA |||| |||||||: :||| CUCC UACUUCGG GGCU<br>- U-- ACCA -	-25
ath-miR167a-5p	3738	A G UCUU A---<br>AGAUCAUGUU GGC GC ||||||||:| ||| || UCUAGUACGA CCG CG<br>A - U--- AAGU	-26.4
ath-miR168a-5p	1547	A -- UGGACU A-<br>UCCUGG UGCACCA GC |||||: ||||||| || AGGGCU ACGUGGU CG<br>A GG U----- CU	-26.6

ath-miR172c	5164	G UA GAA U A<br>CUGCAGC CA CA GAUUCU<br>||||||| || || |||||| GACGUCG GU GU CUAAGA<br>- UA A-- U -	-24.3
ath-miR390a-5p	5148	U AUUA G C A<br>GUGCUAUCUU U CUG AGCU<br>|:||||||:: | ||| |||| CGCGAUAGGG A GAC UCGA<br>C ---- G - A	-26.4
ath-miR824-5p	6380	U A A - AUA A<br>UCC CU UC CACAA GUGGUUUA<br>||| || || ||||| :||||:|| AGG GA AG GUGUU UACCAGAU<br>- - - A --- -	-23.6

Target gene of plant miRNAs: IRS

MIRNA	Position in target gene	Structure of binding site	minimum of free energy
ath-miR156a-5p	1765	A G CCGAUGA C<br>UG UCAUUUUC UCUGUCG<br>|| |||:|:|| ||||||:<br>AC AGUGAGAG AGACAGU<br>C G A------ -	-23
ath-miR157a-5p	4706	C GCG CCGUACG - C-<br>GUGCUC C UAUCU CUGUU<br>|||||| | ||||| ||||:<br>CACGAG G AUAGA GACAG<br>- A-- ------- A UU	-21.3
ath-miR158a-3p	860	G GG GGA --- U<br>GCU UG CUGC UGGGA<br>||| || ||:| ||||| CGA AC GAUG ACCCU<br>A A- A-- UAA -	-20.3
ath-miR160a-5p	859	G - GU CU A-<br>GGC UG GGGA GCUGGG	-27.9

<table>
<tr><td></td><td></td><td> ||| || |||| ||::||
   CCG AU CCCU CGGUCC
  A   U GU   --   GU</td><td></td></tr>
<tr><td>ath-miR162a-3p</td><td>1380</td><td>A   A   U   GAAAAUG   A
 UGGAUG UGG GGU   UGUUGG
 |||||| ::| ||| |:|:|:
 ACCUAC GUC CCA   AUAGCU
G   -   U   A------   -</td><td>-22.9</td></tr>
<tr><td>ath-miR166a-3p</td><td>2497</td><td>C U   -   AGAA A A-
  GG GGAA GAAGC   GG CC
  || |||| ||||| || ||
  CC CCUU CUUCG   CC GG
- -   A   GA-- A CU</td><td>-23.5</td></tr>
<tr><td>osa-miR166g-3p</td><td>1589</td><td>-U   UC-   U   - A-
   GGA   GAG CCUGG CC
   ||| :|| ||||| ||
   CCU   UUC GGACC GG
CU   UAC   -   A CU</td><td>-23.6</td></tr>
<tr><td>ath-miR167a-5p</td><td>841</td><td>-C   U   ACCGG   G--
   GGUCG UGCUGGU   GGCU
   |:||: ||||||:   :|||
   CUAGU ACGACCG   UCGA
AU   -   -----   AGU</td><td>-25.9</td></tr>
<tr><td>ath-miR168a-5p</td><td>1881</td><td>C   -   GAGACGAU UG   G
   UUCC ACCUG   U   CAAGCGA
   |:|| ||||| | |||||||
  AGGG UGGAC   G   GUUCGCU
A   C   -------- UG   -</td><td>-27.9</td></tr>
<tr><td>ath-miR172a</td><td>1340</td><td>-C GU   GU   CAC   G
  G   GCGU   UC   GGAUUCU
  | ||:| || :||||||
  C   CGUA   AG   UCUAAGA
UA GU   GU   U--   -</td><td>-23.7</td></tr>
<tr><td>ath-miR172c</td><td>1340</td><td>-C GU   GU   CAC   G
  G   GCGU   UC   GGAUUCU
  | ||:| || :||||||
  C   CGUA   AG   UCUAAGA</td><td>-23.7</td></tr>
</table>

		GA GU GU U-- -	
ath-miR390a-5p	2905	-C - U CC A-<br>CGC AUC CCUCC GAGC<br>||| ||| ||||| |||| GCG UAG GGAGG CUCG<br>CC A - A- AA	-27
ath-miR397a	1132	G GUUUU A- G<br>AUCA ACGCUGUGC AAUGG<br>|||| |||||::| ||||:<br>UAGU UGCGACGUG UUACU<br>G ----- AG -	-23.6
osa-miR528-5p	1337	U GG ACGGA G<br>UCC UGCGUGUUCC UUCU<br>||| |||:||::|| |||:<br>AGG ACGUACGGGG AAGG<br>G AG ----- U	-26.5
ath-miR824-5p	1747	A GCAUAA - A-<br>UCCUUC UCACA AUGGUC<br>:||||| ||||| |||||| GGGAAG AGUGU UACCAG<br>A ------ U AU	-25.8
ath-miR827	3725	---A AUA - A--<br>UGU UGAUGG CAUC<br>||| |||||| |||| ACA ACUACC GUAG<br>UCAA --- A AUU	-20.3
ath-miR845a	842	G UUGC C G<br>GUCG UGGUA CGGGGCUG<br>:||: ||||| |:|:||:| UAGU ACCAU GUCUCGGC<br>G UA-- A -	-26.1

Target gene of plant miRNAs: EGFR

MIRNA	Position in target gene	Structure of binding site	minimum of free energy

<table>
<tr><td>ath-miR156a-5p</td><td>4761</td><td>-A - GA G-
GUUCGCUC CUU CUGU
|:||:||| ||| ||||
CGAGUGAG GAA GACA
CA A -- GU</td><td>-23</td></tr>
<tr><td>ath-miR157a-5p</td><td>4426</td><td>---A GAUUU C
UCUCUGUCUUC UCAA
|||||:||||| ||||
AGAGAUAGAAG AGUU
CACG AC--- -</td><td>-23.5</td></tr>
<tr><td>ath-miR158a-3p</td><td>2209</td><td>G UGGAAAC AAGGUG U
UGCUUU GUUUACA UUUGGG
|||||| ||:|||| ||||||
ACGAAA CAGAUGU AAACCC
- ------- ------ U</td><td>-21.5</td></tr>
<tr><td>ath-miR160a-5p</td><td>194</td><td>A - U AG C
GGC UGC GGGGAG GGGC
||| |:| :||||| :|||
CCG AUG UCCCUC UCCG
A U - GG U</td><td>-30.8</td></tr>
<tr><td>ath-miR162a-3p</td><td>2897</td><td>U U-- ACGCCGA G
UGGAUGC GG GUCGA
||||||| || :||||
ACCUACG CC UAGCU
G UCU AAA---- -</td><td>-22.8</td></tr>
<tr><td>ath-miR166a-3p</td><td>201</td><td>U A GG AGAACU A A
GGGGAG G GCCUG GUC GA
|||||: | ||||| ||| ||
CCCCUU C CGGAC CAG CU
- A UU ------ G -</td><td>-30.2</td></tr>
<tr><td>osa-miR166g-3p</td><td>201</td><td>U A GG AGAACU A A
GGGGAG G GCCUG GUC GA
|:|||: | ||||| ||| ||
CUCCUU C CGGAC CAG CU
- A UU ------ G -</td><td>-27.2</td></tr>
<tr><td>ath-miR167a-5p</td><td>253</td><td>---C G GGGCAGGU G
UCA UGCUGGCA GCUUCG</td><td>-27.3</td></tr>
</table>

		||| |||||||| |||||:<br>AGU ACGACCGU CGAAGU<br>AUCU - -------- -	
ath-miR168a-5p	2968	A GAUAUCU U AG U<br>UCCUGGCC UGC AUCA GGCGA<br>|||:|:|| ||| |:|| :|||| AGGGCUGG ACG UGGU UCGCU<br>A ------- - -- -	-29.9
ath-miR172a	2020	U C U UAU G<br>UGCGG CAUCAUCA G UUCU<br>|||:| |||||||| | |||| ACGUC GUAGUAGU C AAGA<br>U - U U-- -	-26.2
ath-miR172c	2020	U C U UAU G<br>UGCGG CAUCAUCA G UUCU<br>|||:| |||||||| | |||| ACGUC GUAGUAGU C AAGA<br>G - U U-- -	-26.4
ath-miR390a-5p	270	A UCGGG AA GCGG G-<br>GGUGCU CCC UCC GAGU<br>||:||| ||| ||| |||:<br>CCGCGA GGG AGG CUCG<br>- UA--- -- A--- AA	-27.9
ath-miR397a	5603	A G AUU C--<br>CAUCGA GC UGUAUUCGA<br>||||:| || ||:|:||:| GUAGUU CG ACGUGAGUU<br>- G --- ACU	-23.5
ath-miR403-3p	3263	G GUUGAG AUG C A--<br>GAGUU GU GCG GAAUC<br>||||| || ||| ||||| CUCAA CA CGC CUUAG<br>G A----- --- A AUU	-20.2
osa-miR528-5p	3144	-C - AGUA G<br>UUUCUGCAU CC CUUCCG<br>::||||||| || |||||:<br>GGAGACGUA GG GAAGGU	-27.5

		GA C G--- -	
ath-miR824-5p	2118	C G C C G-<br>CCCUUC C CAA UGG C<br>|||||| | ||| ||| | GGGAAG G GUU ACC G<br>A A U U A AU	-20.8
ath-miR845a	2492	---A ACA UU A<br>AAUUGGUGU CAGA GCC<br>|||||||:| |||| ||| UUAACCAUA GUCU CGG<br>GUAG --- -- C	-23.2

Target gene of plant miRNAs: PDK1

MIRNA	Position in target gene	Structure of binding site	minimum of free energy
ath-miR156a-5p	4978	-C C CUC C U<br>GU CACUCUCU UCU GUCG<br>|: |||||||| ||| |||:<br>CG GUGAGAGA AGA CAGU<br>CA A --- - -	-25
ath-miR157a-5p	5143	-A A GACGCG U A<br>GC UCUU GUC UUCUGUCGA<br>|| |||: :|| |||||||:| CG AGAG UAG AAGACAGUU<br>CA - A----- - -	-24.2
ath-miR160a-5p	4947	G AAA C G U<br>GGCG ACGGGG GC CAGGC<br>|||: ||:||| || ||||| CCGU UGUCCC CG GUCCG<br>A A-- U - U	-33
ath-miR162a-3p	1617	C GUC CUUAGG G<br>CUGGA CGGAG GUCGA<br>||||| |:||| :|||| GACCU GUCUC UAGCU<br>- AC CAAA-- -	-23.2

ath-miR166a-3p	4798	G GG GG AA UU U<br>GGGGG G G UGGU GG<br>||||: | | |||| |:<br>CCCCU C C ACCA CU<br>- UA UU GG GG -	-29.9
osa-miR166g-3p	4798	G GG GG AA UU U<br>GGGGG G G UGGU GG<br>|:||: | | |||| |:<br>CUCCU C C ACCA CU<br>- UA UU GG GG -	-26.9
ath-miR167a-5p	240	-C - GAG G U<br>GAUCG GC GG AGCUUC<br>||||: || || |||||| CUAGU CG CC UCGAAG<br>AU A A-- G U	-26.8
ath-miR168a-5p	896	G G CCCCCUU CUCCAGG G<br>UC CCGGCCUGC CCG AGCGA<br>|| |||:||||| ||: ||||| AG GGCUGGACG GGU UCGCU<br>A - U------ ------- -	-31.3
ath-miR172a	1857	-C A CG G<br>GCA UGUC CGGGAUUC<br>||| ::|| |::||||| CGU GUAG GUUCUAAG<br>UA C UA A	-23.5
ath-miR172c	1857	-C A CG G<br>GCA UGUC CGGGAUUC<br>||| ::|| |::||||| CGU GUAG GUUCUAAG<br>GA C UA A	-23.5
ath-miR390a-5p	993	U AG G CAGUC G<br>GGCG AUCCCUC U GGCU<br>|||| ||||||| | :||| CCGC UAGGGAG A UCGA<br>- GA G C---- A	-27.3
ath-miR397a	1010	A U G G--<br>GUCGGC GCU GUGCUCGA	-24.6

		:||::| ||| |::||||| UAGUUG CGA CGUGAGUU<br>G - - ACU	
ath-miR403-3p	2568	A GAAAAAUA U U<br>CGAG GCGUG AUCUGG<br>|||| ||||| ||||::<br>GCUC CGCAC UAGAUU<br>- AAACA--- U -	-21.6
osa-miR528-5p	899	-G GGC C-- C<br>CC CUGC CCCCUUCCG<br>|| |||| ||||||||:<br>GG GACG GGGGAAGGU<br>GA A-- UAC -	-29.2
ath-miR824-5p	1701	A GAGGGA UUGG G G<br>UCCC UUCGCA AAUGGU UA<br>|||| :||:|| |||||| || AGGG GAGUGU UUACCA AU<br>- AA---- ---- G -	-23.7
ath-miR845a	1005	U CGGCUGC C - A<br>CGUCAGU UGGUG UC GAGCC<br>|:|||:| ||||: || ||||| GUAGUUA ACCAU AG CUCGG<br>- ------- - U C	-25.2

Target gene of plant miRNAs: MKP3

MIRNA	Position in target gene	Structure of binding site	minimum of free energy
ath-miR156a-5p	186	-A UACU C G C<br>GCUC GCUCUCU C UGUU<br>|||| :|||||| | |||:<br>CGAG UGAGAGA G ACAG<br>CA ---- A - U	-22.9
ath-miR157a-5p	193	C CC G G G G<br>UGCUCUCU GU UUC GU GA<br>|||||||| :| ||| || :| ACGAGAGA UA AAG CA UU	-24.3

		C -- G A G -	
ath-miR160a-5p	47	G C CA CU AGA C<br>UGGC UGCAG GG GGCU AGGC |||| |:||| || :||: |||| ACCG AUGUC CC UCGG UCCG<br>- U -- -- --- U	-27.3
ath-miR162a-3p	1679	-----A GACAUUUG U<br>GUAGAGG UAUUGA |:||||| |||:|| CGUCUCC AUAGCU<br>GACCUA AA------ -	-20.6
ath-miR166a-3p	2250	A UGC UUCACU - A<br>GGGAG UGG GUCUGG CCGG ||||: ||: |:|||| |||:<br>CCCUU ACU CGGACC GGCU<br>C --- U----- A -	-27.1
osa-miR166g-3p	2250	A UGC UUCACU - A<br>GGGAG UGG GUCUGG CCGG<br>:|||: ||: |:|||| |||:<br>UCCUU ACU CGGACC GGCU<br>C --- U----- A -	-25.3
ath-miR167a-5p	802	G - AGC GC G<br>AGA CG UGCUGGCAGU CA ||| |: |||||||||: || UCU GU ACGACCGUCG GU<br>A A --- AA -	-26.9
ath-miR168a-5p	45	-G G G U-<br>U UGGCCUGCA CAGGC<br>: :|:|||||| ||:|| G GCUGGACGU GUUCG<br>AA G G CU	-30.5
ath-miR172a	1719	C G - G--<br>UGCAG A CAUCAGGGU |||||	|||||:|:| ACGUC U GUAGUUCUA<br>U G A AGA	-24.1

ath-miR172c	1718	C G - G--<br>CUGCAG A CAUCAGGGU<br>|||||| | |||||:|:| GACGUC U GUAGUUCUA<br>- G A AGA	-25.8
ath-miR390a-5p	2641	-----G A UAU G<br>GUCCCUCCU G GCU<br>:|||||||| | ||| UAGGGAGGA C CGA<br>CCGCGA - U-- A	-25.3
ath-miR397a	427	--A A CUCUGUG A<br>UAACGC GCAU UUAGUGA<br>:||||| |||: |:|:||| GUUGCG CGUG AGUUACU<br>GUA A ------- -	-22.5
ath-miR403-3p	1830	C G--- GG U<br>CGAGU GUGUGAA UAG<br>||||| |:||||| ||:<br>GCUCA CGCACUU AUU<br>- AACA AG -	-21.2
osa-miR528-5p	2645	C A- G--- U<br>CUCCU GUAUGCU CCA<br>||||| |:||||: ||| GAGGA CGUACGG GGU<br>- GA GGAA -	-24.3
ath-miR824-5p	1465	A AAGA C C-<br>CU UCA CAGAUGGUC<br>|: ||| ||:|||||| GG AGU GUUUACCAG<br>A GAAG - AU	-21.2
ath-miR845a	1694	G ACUCUA U U<br>UAUUGAUUGGUG CAGA CC<br>:||::||||||: |||| || GUAGUUAACCAU GUCU GG<br>- A----- C C	-21.1

Target gene of plant miRNAs: PIK3

MIRNA	Position in target gene	Structure of binding site	minimum of free energy
ath-miR167a-5p	346	--U GUA A- A<br>AUCG GC GCAGCUUCA<br>|||: || ||||||||| UAGU CG CGUCGAAGU<br>AUC A-- AC -	-23.8
ath-miR172a	1108	C GU CCA C<br>UGCAG UAUCAUU GAGAUUUU<br>||||| :|||||: :|||||:| ACGUC GUAGUAG UUCUAAGA<br>U -- --- -	-23.2
ath-miR390a-5p	4859	--A C A A-<br>GCU UUCUUCC GAGC<br>||| |:|:||| |||| CGA AGGGAGG CUCG<br>CCG U A AA	-26.3
ath-miR397a	2521	U A A UG U-<br>CGU AACGC UGU UCAAU<br>|:| ||||| ||: ||||| GUA UUGCG ACG AGUUA<br>- G - UG CU	-22.8
osa-miR528-5p	1659	U AG -- A - C<br>CUCC CUGCA CC CCU CCA<br>|||| ||||| || ||| ||| GAGG GACGU GG GGA GGU<br>- A- AC - A -	-26.5
ath-miR824-5p	5498	G AUUA C A---<br>UCUCUUC UACA GUGG<br>||:|||| :||| :||| AGGGAAG GUGU UACC<br>- A--- U AGAU	-20.2
ath-miR845a	5244	--C G C G	-24.4

		CAA UGGUAUUAG GUCG<br>||| ||||||:|| |:|| GUU ACCAUAGUC CGGC<br>GUA A U -	

Target gene of plant miRNAs: PI3K

MIRNA	Position in target gene	Structure of binding site	minimum of free energy
ath-miR156a-5p	5375	-G GUCGGA A<br>GCUCAUUUUU UCUGUCA<br>|||||:|:|: ||||||| CGAGUGAGAG AGACAGU<br>CA A----- -	-27
ath-miR157a-5p	1722	-C CUAUCUC U-<br>GCUCUCUAUCU UCUGUC<br>||||||||||| |||||| CGAGAGAUAGA AGACAG<br>CA ------- UU	-31.2
ath-miR158a-3p	6549	U GG AUCUG ACG A<br>UGUU GUC GCGU GGGA<br>||:| ||| :|:| |||| ACGA CAG UGUA CCCU<br>- AA A---- AA- -	-20.9
ath-miR160a-5p	4738	G - UCCGU CC A<br>UGGCA GCGGG GGCCA GGCA<br>||||| :|:|| :|||| |||| ACCGU UGUCC UCGGU CCGU<br>- A C---- -- -	-31
ath-miR162a-3p	3233	-A C A GC- G<br>G GAUG AGGGG GUCGA<br>| |||| ||:|| :|||| C CUAC UCUCC UAGCU<br>GA - G AAA -	-22.6

ath-miR166a-3p	530	A AGAAAU A C A-<br>GGGAA UGAAG CUUGG CC<br>||||| ||||| |:||| || CCCUU ACUUC GGACC GG<br>C ------ - A CU	-27.4
osa-miR166g-3p	3368	G ACGA CG-- A<br>GAGGAAU GAAGCU CCGA<br>||||||| |||||: |||| CUCCUUA CUUCGG GGCU<br>- ---- ACCA -	-26.2
ath-miR167a-5p	4729	-C U G GGG C<br>GAUCG UG UGGCAGC UC<br>||||: || ||||||| || CUAGU AC ACCGUCG AG<br>AU - G A-- U	-28.3
ath-miR168a-5p	4623	A UU - G<br>UC GGCUUGCAUCGAG GA<br>|| |:|:||||:|:|| || AG CUGGACGUGGUUC CU<br>A GG G -	-32.3
ath-miR172a	6910	C A A-<br>UGCGGCGUUAUCGA GUU<br>|||:||:|:|||:| :|| ACGUCGUAGUAGUU UAA<br>U C GA	-26.1
ath-miR172c	6909	C A A-<br>CUGCGGCGUUAUCGA GUU<br>||||:||:|:|||:| :|| GACGUCGUAGUAGUU UAA<br>- C GA	-27.8
ath-miR390a-5p	927	U AC- A-<br>GCGCUGUUUCU GAGU<br>|||||:|::|| |||:<br>CGCGAUAGGGA CUCG<br>C GGA AA	-26.2
ath-miR397a	3196	A UG G G UU C<br>GUCG GCUG A UU GAUGA	-24.6

<table>
<tr><td></td><td></td><td>:||: |||| | |: :||||
UAGU CGAC U AG UUACU
G   UG   G G --   -</td><td></td></tr>
<tr><td>ath-miR403-3p</td><td>2931</td><td>A   CGAACG -   G--
GAGU   GUG GUGAAUC
|||| ||| |||||||
CUCA   CAC CACUUAG
G   AA---- G   AUU</td><td>-22.3</td></tr>
<tr><td>osa-miR528-5p</td><td>4333</td><td>G   CAGAG C   G G   C
UCCUC   UG CAUGC U CUUCCA
||||| || ||||| : ||||||
AGGAG   AC GUACG G GAAGGU
G   ----- -   - G   -</td><td>-28.2</td></tr>
<tr><td>ath-miR824-5p</td><td>9317</td><td>C   UUCUC   A C-
CCUUUCUCACGA   GUG UC
||:|||||||:| :|| ||
GGGAAGAGUGUU   UAC AG
A   -----   C AU</td><td>-24</td></tr>
<tr><td>ath-miR827</td><td>6547</td><td>-C   --   G
UUUGUUG GGUCAUCUG
||||||| ||||||||:
AAACAAC CCAGUAGAU
UC   UA   U</td><td>-24.5</td></tr>
<tr><td>ath-miR845a</td><td>7744</td><td>-U   A   A-
UCGAUUGGUA UCAGGGU
||:||||||| ||||:|:
AGUUAACCAU AGUCUCG
GU   -   GC</td><td>-27.9</td></tr>
</table>
